# A novel design using a virtual control group to evaluate non-inferiority of nevirapine and lamivudine dual maintenance in HIV therapy

**DOI:** 10.1371/journal.pone.0351576

**Published:** 2026-07-08

**Authors:** Alessandro Suter, Markus Bickel, Carsten Depmeier, Peter Gute, Gaby Knecht, Marcel Stöckle, Christoph Fux, Baharak Babouee Flury, Patrick Schmid, Pietro Vernazza, Christian R. Kahlert

**Affiliations:** 1 Division of Infectious Diseases and Infection Control, Health Ostschweiz (HOCH), Cantonal Hospital St. Gallen, St. Gallen, Switzerland; 2 University of Bern, Berne, Switzerland; 3 Infektiologikum Frankfurt, Frankfurt am Main, Germany; 4 Private practice Wyssgasse, Zurich, Switzerland; 5 Division of Infectious Diseases and Hospital Epidemiology, University Hospital of Basel, Basel, Switzerland; 6 Division of Infectious Diseases, Cantonal Hospital of Aarau, Aarau, Switzerland; 7 Department of Infectious Diseases, Inselspital, Bern University Hospital, University of Bern, Berne, Switzerland; 8 Division of Infectious Diseases and Infection Control, Children’s Hospital of Eastern Switzerland, St. Gallen, Switzerland; University of Perugia Department of Medicine: Universita degli Studi di Perugia Dipartimento di Medicina, ITALY

## Abstract

**Purpose:**

Reducing the number of drugs in combined antiretroviral therapy (cART) likely reduces toxicity. We hypothesized that dual therapy (DT) with nevirapine (NVP) and lamivudine (3TC) would be non-inferior to a virtual control without treatment failure.

**Methods:**

This multicenter study enrolled patients on cART with HIV plasma viral load (pVL) <50 cp/ml for ≥2 years and on NVP for ≥6 months. Patients were compared to a simulated virtual control group with an assumed failure rate of zero. Those with prior Non-Nucleoside Reverse Transcriptase Inhibitor failure or 3TC resistance were excluded. Treatment was simplified to DT with NVP/3TC for 48 weeks, with quarterly pVL-measurements. The primary endpoint was confirmed virologic failure (pVL ≥ 200 cp/mL). A 4% non-inferiority margin and sample size of 201 were set, with a stopping rule if three virologic failures occurred.

**Results:**

From April 2019 to January 2023, 201 patients from five centers in Switzerland and Germany started DT, which 194 participants completed. Two patients (1.03%, 95% CI: –0.92% to 3.68%) reached the primary endpoint for failure due to adherence issues. No additional failures were observed during a 12-month post-study follow-up of 184 participants.

**Conclusions:**

Simplification to NVP and 3TC was as effective as the ideal virtual control. However, the results of NVP and 3TC maintenance therapy are only applicable to people living with HIV who meet the study’s inclusion and exclusion criteria. Virtual controls could improve research efficiency and warrant further evaluation.

## Introduction

The implementation of combined antiretroviral therapy (cART) almost three decades ago has transformed HIV clinical practice by reducing morbidity and mortality. Using three antiretroviral compounds is primarily intended to prevent the development of drug resistance, particularly in the initial phase of treatment when viral replication is high [1]. Due to a markedly reduced likelihood of drug resistance during the maintenance phase of treatment, support for a switch to dual therapy (DT) has increased [1]. Simplifying the medication to two drugs addresses concerns regarding long-term toxicity, patient compliance and the costs of lifelong cART [2]. Recently, Dolutegravir-based (DTG) simplification therapy has received increasing attention [3]. Most of the evidence for this approach is derived from prospective randomised trials in individuals, with no history of treatment failure, who were switched to either DTG/3TC (Lamivudine), DTG/RPV (Rilpivirine) or DTG-FTC (Emtricitabine) [3–6]. Other common non-DTG-combinations were Raltegravir/Darunavir (RAL/DRVb), RAL/ETV (Etravirine) and 3TC/DRVb[7]. All of these studies showed sustained viral suppression similar to continued three-drug treatment, with no evidence of compromised virologic control [7].

For more than two decades, NVP, classified as a non-nucleoside reverse- transcriptase- inhibitor (NNRTI), and 3TC, classified as a nucleoside reverse- transcriptase- inhibitor (NRTI), have been well-known drugs in clinical practice due to their proven long-term tolerability and effectiveness [8]. Both compounds have an outstanding characteristic to penetrate into sanctuaries such as the central nervous system or the genital tract [9]. In addition, NVP is the only NNRTI that does not negatively impact lipid levels and is known to correlate with undetectable HIV-DNA levels [10,11]. Clinical use of NVP has declined in recent years due to a possible serious adverse drug reaction known as hypersensitivity reaction (HSR), which occurs in up to 11% of patients and is characterised by maculopapular rash and/or elevated liver enzymes [12]. It is not recommended as an initial drug in antiretroviral therapy, because high HIV plasma viral load (pVL) has been identified as a risk factor for HSR [13]. HSR is observed within the first few weeks, up to three months after starting the drug [14,15]. However, once full viral suppression is achieved, switching to a NVP-based regimen is associated with a lower risk of HSR independent of the CD4-count at initiation of treatment [14,16]. We recently demonstrated that long-term NVP-based treatment (≥90 days, n = 221) had an overall discontinuation rate of 5.4/100 person years [CI 4.0–7.2] [11]. Patients who have maintained a stable HIV suppression for more than six months on NVP-based cART, may benefit from a combination of NVP and 3TC [15]. An earlier proof-of-concept study showed, that DT with NVP plus 3TC can provide a sufficient HIV maintenance therapy [15].

The purpose of this study was to evaluate a maintenance therapy strategy with NVP plus 3TC in stably treated individuals with chronic HIV infection. We applied a novel approach for a non-inferiority trial design where a new antiretroviral combination therapy is not compared to an existing standard regimen, but instead, the efficacy of this treatment regimen is compared to an ideal virtual control group with a 0% failure rate. This idea was driven by the already excellent clinical results in cART with almost 100% success rate in patients without adherence irregularities, where a new treatment regimen is unlikely to demonstrate superiority over the established standard of care [17]. With such a high level of efficacy, the role of a comparison group in a randomized controlled trial (RCT) needs to be questioned. Choosing a virtual comparison has the potential to considerably reduce study complexity, sample size and cost, thereby helping to conserve valuable resources.

## Methods

### Study design and population

This open label, non-inferiority trial was conducted in five HIV centers in Switzerland (St. Gallen, Aarau, Basel, Zürich) and Germany (Frankfurt). Virological failure (i.e., treatment failure) of an intervention group receiving NVP and 3TC as HIV maintenance therapy was compared to a simulated virtual control with an assumed failure rate of 0%.

Adult participants (>18 years old) on cART including NVP for at least six months with a suppressed HIV viral load (<50 cp/ml) for more than two years were switched to DT with oral NVP and 3TC. Occasional viral blips (i.e., transient HIV-RNA levels of 50–200 cp/ml with subsequent re-suppression to <50 cp/ml without ART modification) were allowed in these two years and didn’t count as an exclusion criterion. Recruitment was conducted from 12^th^ April 2019–24^th^ January 2022. Patients were eligible for the trial, if they had not previously failed any NNRTI based therapy, had no known resistance to NVP or 3TC and had provided written informed consent. Exclusion criteria included chronic Hepatitis B infection (HBs-Ag positive), women who were pregnant or breast feeding, women of childbearing age who were unwilling to use an effective contraception, and any condition, that could interfere with treatment adherence (psychiatric disorders, known adherence problems or health beliefs known to cause patients to discontinue treatment).

### Study intervention and outcome

Study intervention was DT with NVP (400 mg retard tablets) and 3TC (300 mg) in HIV-positive patients on cART maintenance therapy including NVP for at least six months. During the screening phase, patient’s informed consent, demographics, vital signs, HIV-treatment history, laboratory parameters and in-/exclusion-criteria were documented. This was followed by the study period that contained six visits within a time frame of 48 weeks. HIV-RNA measurements were implemented at all study visits every six weeks between baseline and week 12, and every 12 weeks thereafter. All visits included assessment of adherence, concomitant therapies and severe adverse events at baseline, week 6, 12, 24, 36 and 48. Additional clinical and laboratory evaluations were performed according to standard procedures at week 24 and 48. All visits, including HIV-RNA assessments, occurred within a time frame specified in the protocol (S1 File). For organizational purposes, the HIV center in Frankfurt has implemented an expanded timetable, incorporating longer gaps between each participant’s evaluation visits. The entire study period was conducted between 12^th^ April 2019 and 10^th^ January 2023.

The primary endpoint was virological failure, defined as HIV-RNA level ≥200 cp/ml (confirmed by a second measurement within four weeks) during the 48-week study period. In case of an increased HIV pVL ≥ 50 cp/mL, pVL assessment was repeated within four weeks to confirm a viral blip or exclude low-level viral replication. For definitions of safety endpoints and serious adverse events (SAE), we refer to our protocol (S1 File). A protocol defined stopping rule was established to halt the study, if three patients reached the primary endpoint. Additionally, changes in CD4/CD8 cell count were observed in the intervention group to assess changes in immune activation throughout the study period. Individuals, who had no virological failure during the 48-week study period, were given the option to continue DT beyond week 48. In addition, patients were retrospectively followed during a post-study observation period, with HIV-RNA measurements and clinical assessments performed at approximately 6 and 12 months. The post-study surveillance did not include fixed assessment time points and deviations occurred in some cases. The study was conducted in accordance with the study protocol, the latest version of the World Medical Association’s Declaration of Helsinki and all applicable local legal requirements.

### Sample size and statistical methods

In this non-inferiority study, a new regimen was compared with a simulated virtual control group with an assumed 0% failure rate. Based on the 2016 U.S. Food and Drug Administration (FDA) guidance for non-inferiority trials in HIV maintenance studies, a 4% margin for virological failure (primary endpoint) was used [18]. Non-inferiority of the investigational treatment is given, if the upper limit of the two-sided 95% CI for the proportion of virological failure remains below the non-inferiority margin of 4%, indicating that NVP/3TC is no more than 4% less effective than the optimal virtual control (i.e., 0%). A sample size of n = 179 participants in the treatment arm was estimated, assuming a 0% failure rate in the virtual control group, a non-inferiority margin of 4% in the investigational group, a one-sided type I error rate (α) of 0.05 and a power (1 − β) of 0.80. Considering a presumed dropout rate of approximately 10%, the study required a total of 201 patients to be included. In most HIV-maintenance trials, the major reason for failure in both arms are psychiatric comorbidities and other issues affecting adherence. Therefore, it was assumed that an ideal control group with no adherence issues would result in a 100% virological suppression rate.

Virological outcomes were categorized as suggested by the FDA snapshot approach as follows [18]: HIV RNA < 200 cp/ml, HIV-RNA ≥ 200 cp/ml, no virological data (cancelled appointment or on study but not meeting the time window for a scheduled visit). Participants, who announced to withdraw from the study for any reason not related to the treatment (consent withdrawal, relocation, AE or other reasons), were not included in the final analysis as long as their viral load remained completely suppressed at the time of the decision to withdraw from the trial. Upon withdrawal from the study, patients were reverted to a different antiretroviral therapy. To summarize the baseline characteristics, we employed descriptive statistics. For comparison, we simulated data for an ideal control group consisting of a sample of 194 patients with no observed virological failures. All analyses assumed that the sample size (*n*) of the simulated virtual control group was equal to that of the experimental group (194, 184, or 182, depending on the time point). The risk of virological failure under DT was compared to the assumed failure rate in the virtual control group (i.e., 0%) by calculating the risk difference. The Farrington-Manning test as described in Kawasaki et al. (2010) was used to test for non-inferiority (risk difference below 4%), and 95% confidence intervals for the estimated risk difference were obtained by inversion of the Farrington-Manning test [19]. We also evaluated viral blip events using descriptive statistics and changes in the CD4/CD8 ratio over 48 weeks using the Wilcoxon signed-rank test (null hypothesis: median change = 0, α = 0.05). All statistical analyses were conducted using Microsoft Excel and R software (version 4.2.2).

### Ethical approval, consent to participate and inclusivity in global research

This trial was approved by the cantonal ethics committee (Ethikkommission Ostschweiz, EKOS, BASEC-ID: 2019−00215) and registered at the KOFAM (Koordinationsstelle Forschung am Menschen, ID: SNCTP000003395, date of registration: 19/07/2019) an at the US National Library of Medicine (ID: NCT07014189, date of registration: 10/06/2025) [20,21]. All participants provided written informed consent to participate in the study. The study is reported in accordance with the CONSORT guidelines (S2 File). Additional information regarding the ethical, cultural, and scientific considerations specific to inclusivity in global research is included in the Supporting Information (S3 File).

## Results

**Fig 1:**
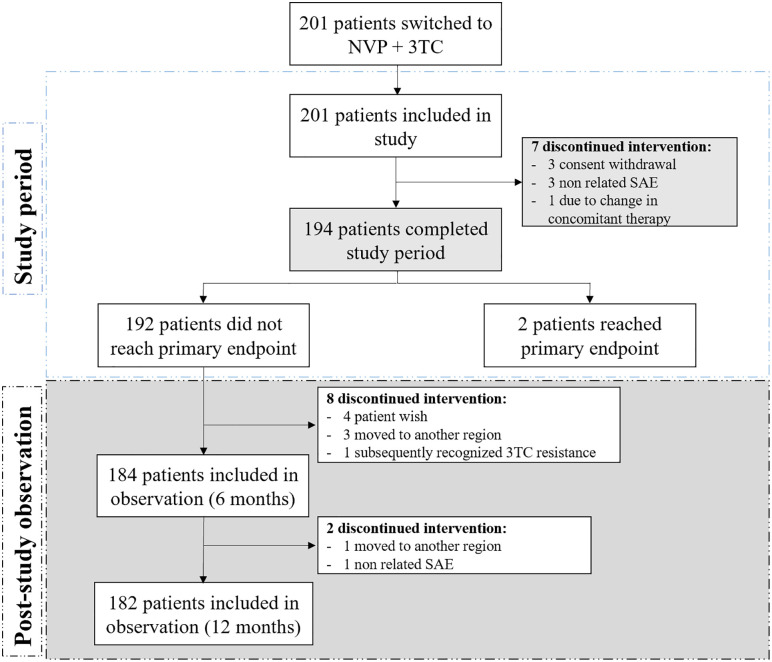
Overview of study participants (flow chart). **NVP:** Nevirapine, **3TC:** Lamivudine, **SAE:** Serious adverse event.

During the study period, a total of 201 patients participated in the study (Fig 1), whereof seven individuals prematurely discontinued participation. Reasons for study discontinuation were withdrawal of consent (n = 3), non-related serious adverse events (SAE, n = 3), change in concomitant medication with a perceived risk of interaction with NVP (n = 1). These seven study participants had a fully suppressed HIV pVL at the time of study discontinuation. 194 patients completed the 48 weeks of the study period. 184 participants continued DT with a predetermined maximum follow-up period of 12 months from September 2020 until May 2024. At baseline, 99% (192/194) of participants had a HIV-RNA below 50 cp/ml. One patient (0.5%) had a viral blip with a confirmatory RNA value <50 cp/ml within four weeks. One other patient (0.5%) had no virological data within the determined time frame, but had suppressed HIV pVL during the screening period. Key baseline and demographic characteristics in the population are presented in Table 1. The median age of the participants was 53 years [IQR 46–60]. Female patients comprised 22% (n = 44) of the group and 13% (n = 26) had a positive Hepatitis C serology. Patients underwent an extended pre-treatment period, with a median duration of 12 years [IQR 8–18] on cART and 65 months [IQR 27–106] on NVP. Median CD4 nadir was 243 cells/µl [IQR 121–350] and the median BMI was at 25 kg/m^2^ [IQR 22–28].

**Table 1:** Baseline characteristics of the population.

Patients included in the non-inferiority trial, n (%)	n = 201 (100%)
**Median age (years) [IQR]**	53 [46-60]
**Gender, n (%)**	
Female	44 (21.9%)
Male	157 (78.1%)
**Positive Hepatitis C serology, n (%)**	26 (12.9%)
**Median CD4 nadir (cells/µl), [IQR]**	243 [121-350]
**Median CD4 absolute (cells/µl), [IQR]**	654 [494-851]
**Median CD4 relative (%), [IQR]**	37 [32 –43]
**Median CD4/CD8 ratio [IQR]**	1.05 [0.75-1.44]
**Median BMI (kg/m** ^**2**^ **) [IQR]**	25.11 [22.15-28.41]
**Median time on ART (years) [IQR]**	12 [8 –18]
**Median time on NVP regimen (months) [IQR]**	65 [27-106]
**CART regimen at inclusion, n (%)**	
NVP-TXF-FTC	101 (50.2%)
NVP-3TC-ABC	97 (48.3%)
Other	3 (1.5%)

**IQR:** Interquartile range, **BMI:** Body Mass Index, **ART:** Antiretroviral therapy, **NVP:** Nevirapine, **CART:** Combined antiretroviral therapy, **TXF:** Tenofovir Disoproxil Fumarate or Tenofovir Alafenamide, **FTC:** Emtricitabine, **3TC:** Lamivudine, **ABC:** Abacavir.

**Fig 2:**
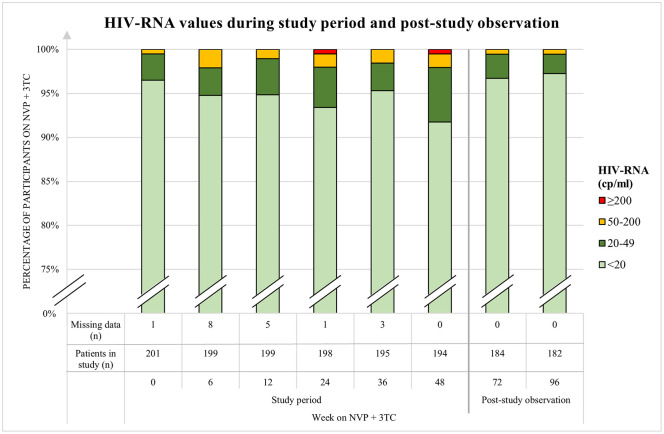
Plasma HIV-RNA for all participants during study period (week 0 until 48) and post-study observation for 1 year (week 72 and 96). **NVP**: Nevirapine, **3TC**: Lamivudine, **RNA**: Ribonucleic acid, **cp/ml:** copies/ml. **Missing data:** Cancelled, postponed, or not yet conducted visit, patient is still included in the study/post-study observation. **HIV-RNA ≥ 200 cp/ml:** Confirmed virological failure. **HIV-RNA ≥ 50-200 cp/ml:** Confirmed viral blip.

192/194 (99%) of the participants maintained full viral load suppression throughout the study period. Two participants (2/194, 1%) reached the primary endpoint of virological failure (two consecutive HIV-RNA ≥ 200 cp/ml) as illustrated in Fig 2. The failure rate (1.03%, 95% CI: –0.92% to 3.68%) was within the pre-specified non-inferiority margin of 4% (z = 2.11, p = 0.017). Detailed medical history of the first patient with virological failure (CD4 nadir 370 cells/µl) revealed a poor drug adherence during a religious fasting period (Ramadan). The second individual with confirmed virological failure (CD4 nadir 146 cells/µl) experienced insufficient adherence to treatment due to a concurrent alcohol use disorder, which worsened during the study period. The first participant’s HIV pVL increased to a peak of 446 cp/ml during the final visit, while the second participant’s pVL rose to 154,000 cp/ml. By the end of the study, the first patient had developed two new mutations (65R and 190A), both of which confer resistance (S1 Table). The second participant had no known new mutations conferring NRTI-resistance. Both patients reached and retained full viral load suppression after changing back to therapy with different components (Darunavir/Ritonavir/Dolutegravir and Doravirin/Lamivudine/Tenofovir disoproxil fumarate respectively). At week 48, virological data was available for all patients. During the study period, a median of 2 participants per visit [IQR 1–4.5] had missing virological data (Fig 2) either due to appointment cancellations or not meeting the time window for a scheduled appointment.

179 of 194 patients (92%) continuously sustained a HIV-RNA < 50 cp/ml throughout 48 weeks (Fig 2 and S1 Fig). Thirteen participants (7%) experienced a total of 16 viral blip events, after which HIV pVL was fully suppressed within a 4-week period following each blip. Among these patients, 23% (3 of 13) self-reported adherence issues. The median CD4 absolute count was 654 cells/µl at baseline and 640 cells/µl at week 48. The CD4/CD8 ratio, an indicator of immune activation and stability in the HIV reservoir, did not change significantly from baseline to week 48 (Wilcoxon signed rank p = 0.80). The median ratio was 1.1 [IQR 0.7–1.4] at baseline and 1.0 [IQR 0.8–1.4] at week 48, with a median change of 0.00 [IQR −0.09 to 0.09; no significant difference from 0].

Among 201 patients, five SAEs were reported (3%) and no SUSARs were observed. Three SAEs (2%) led to an early study termination. One individual experienced a mechanical ileus due to previously known metastatic urothelial carcinoma, that unfortunately resulted in hospitalization and death. Another patient was diagnosed with metastatic lung cancer during a hospitalization, which eventually resulted in death during the study period. The third individual was diagnosed with critical thrombotic occlusion in the left leg, attributed to an advanced peripheral artery disease and testosterone replacement therapy as prothrombotic risk factors. Consequently, a change of the anticoagulant drug regimen (from Dabigatran to Phenprocoumon) had to be established, which lead to discontinuation of NVP due to an increased interaction potential. None of the five SAEs was related to the DT.

Among the 194 patients who reached the 48 weeks of the study, 184 (95%) decided to continue DT thereafter. They were included in the post-study observation. At the time of data curation all patients had completed 12 months of observation (Fig 2). Until the time of the last analysis, no further treatment failure was observed (risk difference after 6 months: 0%, 95% CI: –2.04% to 2.04%, z = 2.77, p = 0.003 and risk difference after 12 months: 0%, 95% CI: –2.07% to 2.07%, z = 2.75, p = 0.003). For 8 of 183 patients (4.5%), 12-month data were unavailable, 18-month data were used instead. Since these patients remained virologically suppressed over the longer follow-up period, they were assumed to have been suppressed at 12 months. Eight patients discontinued DT within the first six months whereof three individuals moved to another city. Four patients ceased the treatment due to personal reasons. One individual changed therapy regimen to another cART due to a pre-existing drug resistance against 3TC, which was only discovered after the study period ended. During the second phase of the post-study observation, which spanned from 6 to 12 months, two additional patients stopped DT. One patient relocated to another region in Switzerland, while another discontinued cART because of a non-related SAE (previously known lingual carcinoma with continuous deterioration of general condition, which ultimately ended in death). All patients had fully suppressed HIV-RNA at the time of discontinuation. The proportion of participants remaining in the study and post-study observation is illustrated in Figs 1 and 2.

## Discussion

In this trial, the efficacy of DT with NVP/3TC was compared to a virtual ideal control group without treatment failure (i.e. cART). The results demonstrated non-inferiority for the primary endpoint of virological failure. A considerable proportion of the observed population maintained virological suppression (99%). Only two individuals (1%) reached the primary endpoint during the study period. During the post-study observation period, 94% of participants (182 out of 194) continued DT for at least 12 months without any occurrence of treatment failure.

This two-drug regimen demonstrates potential as an HIV maintenance therapy for patients currently on NVP based cART. It has shown to be both effective and safe in patients who have been taking NVP for more than six months. The regimen offers a promising alternative for long-term HIV management, potentially simplifying treatment while maintaining viral suppression. No AIDS defining events have been registered during the study period. Furthermore, the long-term safety of both drugs is well-established and cost-effective. Compared to other DT regimens, this regimen achieved similar efficacy in sustaining low HIV pVL [5,7]. Recent research has focused on DT containing DTG, which has demonstrated long-term efficacy in maintaining virologic suppression without the risk of drug resistance in several large trials [5,22]. The majority of these studies were RCT’s and observational studies with an overall virological failure rate of 0.7% [5]. Our trial demonstrated comparable efficacy in controlling viral load. However, it is important to note that the comparison with an ideal control likely underestimates the true efficacy of the treatment. This conservative approach potentially yields in results that are more robust than those obtained through conventional comparative methods. According to our current knowledge, this therapy has no known long-term side effects [23]. Despite NVP having a low resistance barrier and requiring only a single mutation in the reverse transcriptase genome to develop resistance [15], DT with 3TC proved to be an efficient combination to maintain viral suppression. This observation suggests, that the effective compartment penetration of NVP and 3TC may explain the lower likelihood of virological failure compared to using a single compound with a high barrier against drug resistance (such as DTG) [24–26]. Based on these findings, we might hypothesize that a high infiltration into viral sanctuaries could be as crucial as a strong genetic barrier to resistance [15]. This hypothesis is further supported by the correlation between NVP use and undetectable proviral HIV-DNA [27] as well as the stable CD4 count and CD4/CD8 ratio observed in this study, which indicates no substantial increase in immune activation. Several studies have suggested that a consistent CD4/CD8 ratio, in addition to serving as a risk estimation for non-AIDS events like cancer and cardiovascular events, could also serve as a predictor for stable HIV reservoir levels [28]. In high-income countries, NVP has become less frequently used in clinical practice due to its increased risk of HSR during the initiation phase. However, multiple studies have demonstrated that the risk of HSR decreases greatly, if the medication is introduced when the viral load is undetectable [12,13]. Especially for patients on stable NVP-based cART, NVP likely represents a legitimate option as a long term drug in DT and part of standard maintenance cART [11]. Given that NVP and 3TC are widely available as generics, they offer high cost-saving potential.

We used a novel strategy to assess efficacy of maintenance therapy with NVP and 3TC by comparing the intervention group with a simulated virtual control with the assumption of a 100% viral suppression rate. In the context of modern cART, if patients who intentionally discontinue ART or those with a considerable psychiatric comorbidity are excluded from studies, it is highly likely that a viral suppression rate of 100% could be achieved. Consequently, an ideal control group may be postulated for comparison purposes. In a non-inferiority trial with a 4% non-inferiority margin in the intervention group, the choice of an idealized control ensures that treatment efficacy is not overestimated, but at best underestimated. When applying a highly effective therapy regimen like cART, it becomes crucial to question the necessity of a real comparison group in a RCT. Despite being widely accepted as the “gold standard”, randomized designs for evaluating medical interventions face challenges such as ethical issues, insufficient sample sizes and a high expenditure of resources [29]. Compared to the randomized approach, the strategy of a virtual control group offers several advantages in a non-inferiority trial for HIV maintenance therapy. They ensure equal access to the investigated treatment for all participants, and provide great cost-effectiveness and efficiency, reducing the need for additional recruitment and data collection [29]. The removal of blinding and randomization allows study participants to know their allocated treatment, which may reduce the risk of loss to follow-up due to a perceived disadvantage to other participants. Finally, the study design allows a substantial decrease of the sample size for the same statistical power [29]. Serving as a reference for cART without treatment failure (i.e., a treatment with the best possible outcome), virtual control groups can simplify the evaluation of non-inferiority in new HIV regimens and may offer a viable alternative to traditional randomized controlled trial designs.

There are several limitations to this study. In a virtual control group, the reliance on existing data may result in missing or incomplete outcome information, which can impact the accuracy of the trial results [29]. For example, safety endpoints are missing in the simulated virtual control group (e.g., lipid levels), thereby we cannot confirm the statement that two drugs are safer than three drugs. However, there was no relevant change in adverse event rates when HIV-therapy was simplified from a triple to a dual-drug therapy. As an example DTG-based simplification did not result in increased adverse events [3–5]. Additionally, the lack of randomization increases the risk for selection bias [29]. A limitation of this trial is the selection of a 4% non-inferiority margin, as the FDA does not explicitly support this threshold for comparisons involving a virtual ideal control. We adhered to the general FDA guidelines on non-inferiority trials for HIV maintenance therapy and applied them in this study [30]. Another limitation to consider is the potential for overestimating adverse events in the intervention group while comparing SAEs to a perfect virtual therapy. The results of NVP and 3TC maintenance therapy can only be applied to a certain population of HIV patients, that matches the inclusion and exclusion criteria of this trial. For example in people on unstable cART regimen or with concomitant chronic Hepatitis B, a switch to this drug regimen might provide insufficient treatment and therefore, a three drug regimen with the inclusion of Tenofovir disoproxil fumarate/Tenofovir alafenamide/FTC should be continued [1]. The limited diversity of ethnicity, sex, and age in the study group restricts generalizability and the strategy requires confirmation in other populations such as children, young adult men and women. Additionally, 48 weeks and approximately 12 months post-study observation still is a short time period to assess efficacy of this DT. A prolonged follow-up over several years is required to evaluate long-term effectiveness. Along with long-term CD4/CD8 ratio, another important aspect is to obtain a more detailed observation of immune activation markers such as human leukocyte antigen (HLA)-DR and CD38 antigen on CD8 + T-lymphocytes to detect potential active viral replication. To further assess this treatment, follow-up for long-term effectiveness and safety with assessment of changes in virus reservoir (HIV-DNA) and immune activation is needed. Follow-up studies on these topics are currently being considered.

In conclusion, dual HIV maintenance therapy with NVP and 3TC provided significant, continuous suppression of HIV pVL and was non-inferior to a virtual ideal control with a 0% failure rate. Treatment failures only occurred when the drugs were not taken regularly. This combination can be considered as an alternative to other two-drug regimens. However, it is only applicable to people living with HIV who meet the study’s inclusion and exclusion criteria. The use of a virtual control group to assess a two drug ART may represent a novel approach, which could potentially simplify research in HIV maintenance therapy but needs further assessment.

## Supporting information

S1 Table Genotypic drug resistance interpretation (HIV-1) of one participant with virological failure. **White: No resistance; light grey: Pre-existing resistance; dark grey: New resistance. NRTI:** Nucleoside/Nucleotide Reverse Transcriptase Inhibitor, **NNRTI:** Non-Nucleoside Reverse Transcriptase Inhibitor, **PI:** Protease Inhibitors, **INSTI:** Integrase Strand Transfer Inhibitors**. NVP**: Nevirapine, **3TC**: Lamivudine, **FTC:** Emtricitabine, **ABC:** Abacavir, **TDF/TAF:** Tenofovir, **ZDV:** Zidovudine, **DOR:** Doravirine, **EFV:** Efavirenz, **ETR:** Etravirine, **RPV:** Rilpivirine, **ATV:** Atazanavir/Ritonavir, **DRV:** Darunavir/Ritonavir, **LPV/r:** Lopinavir, **TPV:** Tipranavir/Ritonavir, **BIC:** Bictegravir, **CAB:** Cabotegravir, **DTG:** Dolutegravir, **EVG:** Elvitegravir, **RAL:** Raltegravir. **Mutations list Reverse Transcriptase (RT):** 65R, 190A. **Mutations list Protease (PR):** 10l, 11l, 36l, 69Q, 89M.
(PDF)

S1 Fig Proportion of patients on NVP and 3TC per week. **NVP**: Nevirapine, **3TC**: Lamivudine, **S(t):** Proportion of participants who have not discontinued treatment at time t.
(TIFF)

S1 File Study protocol. (PDF)

S2 File Consort checklist. (PDF)

S3 File Inclusivity in global research questionnaire. (PDF)
