# Supplementary material for: A novel design using a virtual control group to evaluate non-inferiority of nevirapine and lamivudine dual maintenance in HIV therapy
Source: PLoS One. 2026 Jul 8;21(7):e0351576. doi: 10.1371/journal.pone.0351576 (PMC13345260; doi:10.1371/journal.pone.0351576)
Supplement: S1 Table — White: No resistance; light grey: Pre-existing resistance; dark grey: New resistance. NRTI: Nucleoside/Nucleotide Reverse Transcriptase Inhibitor, NNRTI: Non-Nucleoside Reverse Transcriptase Inhibitor, PI: Protease Inhibitors, INSTI: Integrase Strand Transfer Inhibitors. NVP: Nevirapine, 3TC: Lamivudine, FTC: Emtricitabine, ABC: Abacavir, TDF/TAF: Tenofovir, ZDV: Zidovudine, DOR: Doravirine, EFV: Efavirenz, ETR: Etravirine, RPV: Rilpivirine, ATV: Atazanavir/Ritonavir, DRV: Darunavir/Ritonavir, LPV/r: Lopinavir, TPV: Tipranavir/Ritonavir, BIC: Bictegravir, CAB: Cabotegravir, DTG: Dolutegravir, EVG: Elvitegravir, RAL: Raltegravir. Mutations list Reverse Transcriptase (RT): 65R, 190A. Mutations list Protease (PR): 10l, 11l, 36l, 69Q, 89M. (PDF) [file pone.0351576.s001.pdf]

S1 Table. Genotypic drug resistance interpretation (HIV-1) of one participant with virological failure.

Category	Drug	Mutations list	Range	Interpretation
NRTI	3TC/FTC	65R	3	NR – New Resistance
NRTI	ABC	65R	3	NR – New Resistance
NRTI	TDF/TAF	65R	3	NR – New Resistance
NRTI	ZDV		1	S - Susceptible
NNRTI	DOR	190A	1	S - Susceptible
NNRTI	EFV	190A	3	NR – New Resistance
NNRTI	ETR	190A	1	S - Susceptible
NNRTI	NVP	190A	3	NR – New Resistance
NNRTI	RPV		1	S - Susceptible
PI	ATV	10I	1	S - Susceptible
PI	DRV	11I	1	S - Susceptible
PI	LPV/r	10I	1	S - Susceptible
PI	TPV	36I, 69Q, 89M	3	R - Resistance
INSTI	BIC		1	S - Susceptible
INSTI	CAB		1	S - Susceptible
INSTI	DTG		1	S - Susceptible
INSTI	EVG		1	S - Susceptible
INSTI	RAL		1	S - Susceptible
