# Supplementary figures and images for: A novel design using a virtual control group to evaluate non-inferiority of nevirapine and lamivudine dual maintenance in HIV therapy
Source: PLoS One. 2026 Jul 8;21(7):e0351576. doi: 10.1371/journal.pone.0351576 (PMC13345260; doi:10.1371/journal.pone.0351576)

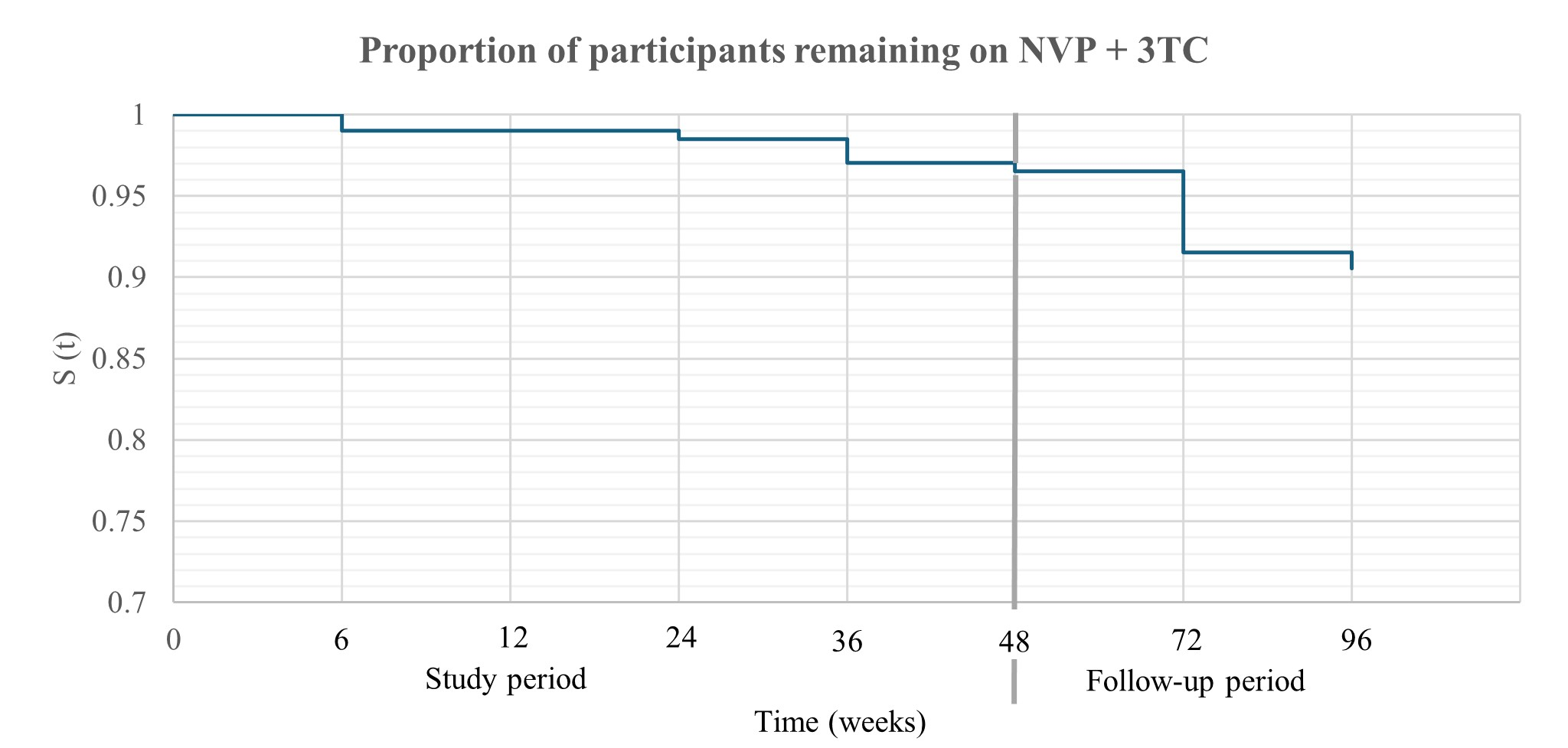

Supplement: S1 Fig — NVP: Nevirapine, 3TC: Lamivudine, S(t): Proportion of participants who have not discontinued treatment at time t. (TIFF) [file pone.0351576.s002.tiff]
