# Supplementary material for: A novel design using a virtual control group to evaluate non-inferiority of nevirapine and lamivudine dual maintenance in HIV therapy
Source: PLoS One. 2026 Jul 8;21(7):e0351576. doi: 10.1371/journal.pone.0351576 (PMC13345260; doi:10.1371/journal.pone.0351576)
Supplement: S1 File — (PDF) [file pone.0351576.s003.pdf]

Open-label multicenter non-inferiority trial of Nevirapine plus Lamivudine versus optimal standard in HIV maintenance therapy

Short Title: Nevirapine / Lamivudine for HIV maintenance therapy

Clinical Study Protocol

Study Type:	Investigator initiated clinical trial with Nevirapine / Lamivudine bi-therapy (Investigational Medicinal Products)
Study Categorisation:	Risk category A
Study Registration:	Clinicaltrials.gov (intended)
Study Identifier:	CTU 17/027
Sponsor, Sponsor-Investigator or Principal Investigator:	Sponsor: Cantonal Hospital St. Gallen, Switzerland<br>Sponsor-Investigator: Pietro Vernazza, Clinic / Division of Infectious Diseases and Hospital Epidemiology<br>Rorschacherstrasse 95, Cantonal Hospital St. Gallen, St, Gallen<br>T: +41 71 494 2631<br>M: pietro.vernazza@kssg.ch
Investigational Product:	Nevirapine (Viramune®), Lamivudine (3TC®)
Protocol Version and Date:	Version 1.0, February 4th 2019

CONFIDENTIAL

The information contained in this document is confidential and the property of the sponsor. The information may not - in full or in part - be transmitted, reproduced, published, or disclosed to others than the applicable Competent Ethics Committee(s) and Regulatory Authority(ies) without prior written authorisation from the sponsor except to the extent necessary to obtain informed consent from those who will participate in the study.

Signature Page(s)

Study number	CTU 17/027
Study Title	Open-label multicenter non-inferiority trial of Nevirapine plus Lamivudine versus optimal standard in HIV maintenance therapy

The Sponsor-Investigator and trial statistician have approved the protocol version 1.0 [04/02/2019], and confirm hereby to conduct the study according to the protocol, current version of the World Medical Association Declaration of Helsinki, ICH-GCP guidelines or ISO 14155 norm if applicable and the local legally applicable requirements.

Sponsor-Investigator: Pietro Vernazza

Place/Date	Signature

Local Principal Investigator at study site St. Gallen:

I have read and understood this trial protocol and agree to conduct the trial as set out in this study protocol, the current version of the World Medical Association Declaration of Helsinki, ICH-GCP guidelines or ISO 14155 norm and the local legally applicable requirements.

Site	Clinic / Division of Infectious Diseases, Cantonal Hospital St. Gallen
Principal investigator	Christian Kahlert

Place/Date Signature

Local Principal Investigator at study site Aarau:

I have read and understood this trial protocol and agree to conduct the trial as set out in this study protocol, the current version of the World Medical Association Declaration of Helsinki, ICH-GCP guidelines or ISO 14155 norm and the local legally applicable requirements.

Site	Division of Infectious Diseases and Hospital Epidemiology, Cantonal Hospital Aarau
Principal investigator	Christoph Fux

Place/Date Signature

Local Principal Investigator at study site Basel:

I have read and understood this trial protocol and agree to conduct the trial as set out in this study protocol, the current version of the World Medical Association Declaration of Helsinki, ICH-GCP guidelines or ISO 14155 norm and the local legally applicable requirements.

Site	Department of Infectious Diseases & Hospital Hygiene, University Hospital Basel
Principal investigator	Marcel Stöckle

Place/Date Signature

Local Principal Investigator at study site Zürich:

I have read and understood this trial protocol and agree to conduct the trial as set out in this study protocol, the current version of the World Medical Association Declaration of Helsinki, ICH-GCP guidelines or ISO 14155 norm and the local legally applicable requirements.

Site	Arztpraxis Kalkbreite Zürich
Principal investigator	Carsten Depmeier

Place/Date Signature

Trial Statistician	Andrea Bregenzer

____________________ ______________________________

Place/Date Signature

Table of Contents

STUDY SYNOPSIS 8
STUDY SUMMARY IN LOCAL LANGUAGE 10
ABBREVIATIONS 11
STUDY SCHEDULE 12
1. STUDY ADMINISTRATIVE STRUCTURE 13
1.1 Sponsor, Sponsor-Investigator 13
1.2 Principal Investigator(s) 13
1.3 Statistician ("Biostatistician") 14
1.4 Laboratory 14
1.5 Monitoring institution 14
1.6 Data Safety Monitoring Committee 14
1.7 Any other relevant Committee, Person, Organisation, Institution 14
2. ETHICAL AND REGULATORY ASPECTS 15
2.1 Study registration 15
2.2 Categorisation of study 15
2.3 Competent Ethics Committee (CEC) 15
2.4 Competent Authorities (CA) 15
2.5 Ethical Conduct of the Study 15
2.6 Declaration of interest 15
2.7 Patient Information and Informed Consent 15
2.8 Participant privacy and confidentiality 16
2.9 Early termination of the study 16
2.10 Protocol amendments 16
3. BACKGROUND AND RATIONALE 17
3.1 Background and Rationale 17
3.1.1 Standard therapy for HIV 17
3.1.2 Monotherapy in the maintenance phase 17
3.1.3 Monotherapies with dolutegraivr (DTG) 17
3.1.4 Nevirapine as an underestimated antiretroviral drug 18
3.1.5 Evaluation of new HIV regimens 18
3.1.6 Own Experience in the Field 19
3.2 Investigational Product (treatment, device) and Indication 20
3.3 Preclinical Evidence 20
3.4 Clinical Evidence to Date 20
3.5 Dose Rationale / Medical Device: Rationale for the intended purpose in study (pre-market MD) 20
3.6 Explanation for choice of comparator (or placebo) 20
3.7 Risks / Benefits 20
3.8 Justification of choice of study population 21
4. STUDY OBJECTIVES 22
4.1 Overall Objective 22
4.2 Primary Objective 22
4.3 Secondary Objectives 22
4.4 Safety Objectives 22
5. STUDY OUTCOMES 23
5.1 Primary Outcome 23
5.2 Secondary Outcomes 23
5.3 Other Outcomes of Interest 23
5.4 Safety Outcomes 23

6. STUDY DESIGN 24
6.1 General study design and justification of design 24
6.2 Methods of minimising bias 24
6.2.1 Randomisation 24
6.2.2 Blinding procedures 24
6.2.3 Other methods of minimising bias 24
6.3 Unblinding Procedures (Code break) 24
7. STUDY POPULATION 25
7.1 Eligibility criteria 25
7.2 Recruitment and screening 25
7.3 Assignment to study groups 25
7.4 Criteria for withdrawal / discontinuation of participants 25
8. STUDY INTERVENTION 26
8.1 Identity of Investigational Products (treatment / medical device) 26
8.1.1 Experimental Intervention (treatment / medical device) 26
8.1.2 Control Intervention (standard/routine/comparator treatment / medical device) 26
8.1.3 Packaging, Labelling and Supply (re-supply) 26
8.1.4 Storage Conditions 26
8.2 Administration of experimental and control interventions 26
8.2.1 Experimental Intervention 26
8.2.2 Control Intervention 26
8.3 Dose modifications 26
8.4 Compliance with study intervention 26
8.5 Data Collection and Follow-up for withdrawn participants 26
8.6 Trial specific preventive measures 27
8.7 Concomitant Interventions (treatments) 27
8.8 Study Drug Accountability 27
8.9 Return or Destruction of Study Drug 27
9. STUDY ASSESSMENTS 28
9.1 Table of study procedures and assessments 28
9.2 Assessments of outcomes 28
9.2.1 Assessment of primary outcome 28
9.2.2 Assessment of secondary outcomes 29
9.2.3 Assessment of other outcomes of interest 29
9.2.4 Assessment of safety outcomes 30
9.2.5 Assessments in participants who prematurely stop the study 30
9.3 Procedures at each visit 30
9.3.1 Visit 1 / Screening visit (week -4 to 0) 30
9.3.2 Visit 2, Baseline (sampling for HIV-1 DNA quantification and immune activation markers) 30
9.3.3 Scheduling of next visitVisit 3,4, and 6 (sampling for HIV-RNA quantification) 30
9.3.4 Visit 5(week 24; sampling for HIV-RNA quantification and clinical follow-up) 31
9.3.5 Visit 7 (week 48; sampling for quantification of HIV-RNA-, HIV-1 DNA- and immune activation markers and clinical follow-up) 31
10. SAFETY 32
10.1 Drug studies 32
10.1.1 Definition and assessment of (serious) adverse events and other safety related events 32
10.1.2 Reporting of serious adverse events (SAE) and other safety related events 33
10.1.3 Follow up of (Serious) Adverse Events 33
11. STATISTICAL METHODS 34
11.1 Hypothesis 34

11.2 Determination of Sample Size ... 34
11.3 Statistical criteria of termination of trial ... 34
11.4 Planned Analyses ... 34
11.4.1 Datasets to be analysed, analysis populations ... 34
11.4.2 Primary Analysis ... 34
11.4.3 Secondary Analyses ... 36
11.4.4 Interim analyses ... 36
11.4.5 Safety analysis ... 36
11.4.6 Deviation(s) from the original statistical plan ... 36
11.5 Handling of missing data and drop-outs ... 36
12. QUALITY ASSURANCE AND CONTROL ... 37
12.1 Data handling and record keeping / archiving ... 37
12.1.1 Case Report Forms ... 37
12.1.2 Specification of source documents ... 37
12.1.3 Record keeping / archiving ... 37
12.2 Data management ... 37
12.2.1 Data Management System ... 37
12.2.2 Data security, access and back-up ... 37
12.2.3 Analysis and archiving ... 37
12.2.4 Electronic and central data validation ... 37
12.3 Monitoring ... 38
12.4 Audits and Inspections ... 38
12.5 Confidentiality, Data Protection ... 38
12.6 Storage of biological material and related health data ... 38
13. PUBLICATION AND DISSEMINATION POLICY ... 39
14. FUNDING AND SUPPORT ... 39
14.1 Funding ... 39
14.2 Other Support ... 39
15. INSURANCE ... 39
16. REFERENCES ... 40
17. APPENDICES ... 42

STUDY SYNOPSIS

**Sponsor / Sponsor-Investigator**	Cantonal Hospital St. Gallen / Pietro Vernazza
**Study Title:**	Open-label multicenter non-inferiority trial of Nevirapine plus Lamivudine versus optimal standard in HIV maintenance therapy
**Short Title / Study ID:**	Neviparine (NVP)/ Lamivudine (3TC) for HIV maintenance therapy
**Protocol Version / Date:**	Version 1.0 / February 4th 2019
**Trial registration:**	Clinicaltrials.gov (intended)
**Study category and Rationale**	A<br>According to Art. 19 ClinO Paragraph 4, Category A is justified due to comprehensive safety data available for the investigational medicinal products both of which have been authorized in Switzerland for HIV therapy for years.
**Clinical Phase:**	Clinical phase 4
**Background and Rationale:**	Treatment of HIV infection requires lifelong medication. Reducing drug exposure has the potential to limit costs and side effects. Monotherapy with ritonavir boosted protease inhibitors or with integrase inhibitors have been conducted with reasonable short term (48 weeks) efficacy in approx. 80% of treated individuals. Combination of NVP and lamivudine has the potential of very limited adverse events and costs. A local pilot study at the KSSG demonstrated no blip or virological failure (HIV-RNA >50 cp/ml) in 20 patients over more than 1300 observation weeks.
**Objective(s):**	Demonstrate that NVP/lamivudine maintenance is non-inferior to an ideal (virtual) standard therapy with 0% failure rate (i.e. best possible result in a non-inferiority study) and exclude (as secondary objectives) other indications for reduced antiretroviral activity of the bi-therapy.
**Outcome(s):**	Primary:<br>Proportion of patients with HIV-RNA ≥ 200 cp/ml at week 48 (confirmed by a second measurement within 4 weeks)<br>Secondary:<br>a) Increase in frequency of low level HIV-RNA detection<br>b) Increase in HIV reservoir size<br>c) Increase in immune activation markers
**Study design:**	Open label, multicentre non-inferiority trial comparing NVP plus lamivudine with a virtual control in HIV maintenance therapy.
**Inclusion / Exclusion criteria:**	Inclusion criteria:<br>1. >18 Years old<br>2. Patients on a stable HIV-therapy containing NVP for at least six months<br>3. Duration of viral load suppression (HIV-RNA < 50 cp/ml) of more than 2 years (allowing occasional blips, i.e. viral load measurements of 50-200 HIV-RNA cp/ml followed by an additional measurement of <50 cp/ml within 4 weeks)<br>4. No previous failure of any NNRTI based therapy<br>5. No known resistance to NVP or 3TC<br>6. Able to fully understand the informed consent and the experimental nature of the study<br>7. Absence of any health believe systems that might interfere with drug intake<br>Exclusion criteria<br>I. Chronic hepatitis B infection (HBs-AG positive)<br>II. Any condition, that might, at the discretion of the principle investigator, interfere with treatment adherence or regular HIV-RNA testing appointments, such as<br>i) psychiatric disorders,<br>ii) known adherence problems in the past 2 years or<br>iii) health believe issues known to cause patients to stop treatment (e.g. expecting God to cure HIV or not believing the existence of HIV, etc.)

**Measurements and procedures:**	Intervention:<br>- Treatment with nevirapine (400mg retard) and lamivudine (300mg) once daily<br>Clinical procedures:<br>- Assessment of adherence, concomitant therapies and severe adverse events that might have occurred during the study at week 6, 12, 24, 36 and 48.<br>Laboratory procedures:<br>- Viral load measurement every 6 weeks up to week 12, then every 12 weeks up to week 48<br>- HIV-1 DNA measurement at week 0 and 48<br>- Measurement of immune system activation markers (HLA-DR+ and CD38+ on CD4+, CD8+and CD3+ T-lymphocytes) at week 0 and 48 (anticipated for site in St. Gallen only)
**Study Product / Intervention:**	Patients on stable NVP treatment in combination with one or two NRTI will continue with a bi-therapy consisting of nevirapine and lamivudine.<br>Standard dosing of nevirapine (400mg retard once daily) and lamivudine (300mg once daily) will be used.
**Control Intervention (if applicable):**	Not applicable. Intervention is compared to an idealized virtual arm of standard therapy with an assumed 0% virological failure rate
**Number of Participants with Rationale:**	n = 200 (total)<br>Sample size calculation is based on the assumption of a 0% failure rate in the virtual control arm and a 4% non-inferiority margin for the investigational arm.
**Study Duration:**	24 months (April 2019 – March 2021)
**Study Schedule:**	First-Participant-In: April 2019 (planned)<br>Last-Participant-In: March 31st, 2020<br>First-Participant-Out: March 31st, 2020<br>Last-Participant-Out: March 31st 2021 (planned)
**Investigator(s):**	Pietro Vernazza and Christian Kahlert, Infektiologie, Kantonsspital St. Gallen<br>Andrea Bregenzer and Christoph Fux, Kantonsspital Aarau<br>Carsten Depmeier, Kalkbreite Praxis, Zürich<br>Marcel Stöckle, Universitätsspital Basel<br>See contact details in Section 1.2 on page 13
**Study Centre(s):**	Multi-centre: the study will be conducted at 4 centres (Kantonspital St. Gallen, Kantonsspital Aarau, Universitätsspital Basel, Praxis Kalkbreite Zürich).
**Statistical Considerations:**	Based on the experience of long-term maintenance of HIV treatment, and the preliminary data of our pilot study, we expect a failure rate of <1%. Two virological failures in 179 patients would result in a failure rate of 1.11% (95% CI: 0.31-3.98%). Comparing this failure rate to 0% in the virtual control group, non-inferiority (with a non-inferiority margin of 4%) is shown (upper limit of the 95% confidence-interval remains below 4%).
**GCP Statement:**	This study will be conducted in compliance with the protocol, the current version of the Declaration of Helsinki, the ICH-GCP or ISO EN 14155 (as far as applicable) as well as all national legal and regulatory requirements.

STUDY SUMMARY IN LOCAL LANGUAGE

Die HIV-Infektion wird standardmässig immer mit einer Kombination von drei HIV-Medikamenten behandelt (Günthard 2014). Der Grund für die Kombination mehrerer Substanzen ist vor allem das rasche Auftreten von Resistenzen, wenn nur mit einer oder zwei Substanzen behandelt wird. In den letzten Jahren hat sich jedoch gezeigt, dass die Gefahr einer Resistenzbildung deutlich geringer ist, ist die Virusvermehrung im Körper einmal vollständig unterdrückt. Für diese sogenannte Therapie-Erhaltungsphase wurden nun Studien durchgeführt, welche versuchen, die Anzahl der Medikamente zu reduzieren.

Es gibt einige recht gute Erfahrungen mit Monotherapie. Einerseits mit Protease-Hemmern (1), neuerdings auch mit Integrase-Hemmern (2,3). Allerdings zeigen beide Wirkstoffgruppen ein Versagen der Monotherapie bei ca. 10-20% der Patienten. Wir vermuten dass die schlechte Penetration in den Genitaltrakt und ins Gehirn für dieses Resultat mitverantwortlich ist (4). Im Gegensatz dazu zeigen neuere Studien zur Zweierkombination von Protease- oder Integrase-Hemmern mit Lamivudin eine sehr gute Wirksamkeit (5–7). Von Lamivudin wissen wir, dass es sehr gut im Gehirn und im Genitaltrakt wirkt. Da die HIV-Medikation praktisch lebenslang eingenommen werden muss (8), ist eine Vereinfachung der Therapie von grosser Bedeutung. Sie reduziert die potentiell immer möglichen Langzeit-Nebenwirkungen, und sie reduziert auch Kosten.

Die Klinik für Infektiologie/Spitalhygiene am KSSG hat langjährige Erfahrung mit einem bereits vor über 20 Jahren eingeführten HIV-Medikament: Nevirapine. Gut ein Drittel der Patienten wird mit dieser Substanz behandelt. Von den zusätzlichen in Kombination mit Nevirapin verwendeten Präparaten enthalten beide Lamivudin (oder das analoge Emtricitabin) plus entweder Abacavir oder Tenofovir. Beide letztgenannten haben potentiell relevante Langzeit-Nebenwirkungen (Kardiovaskuläres Risiko resp. Nieren-/Knochen-Toxizität).

Eine Zweierkombination mit Nevirapin und Lamivudin hat erstens eine grosse Chance wirksam zu sein. Zweitens ist sie von allen alternativen Kombinationsmöglichkeiten die günstigste. Nach allem was wir heute wissen, hat diese Kombination auch keine bekannten Langzeit-Nebenwirkungen.

Nach Durchführung einer kontrollierten Pilot-Studie mit 20 Patienten am KSSG verfügen wir nun über Daten, welche die Kombination von Nevirapin und Lamivudin zur Aufrechterhaltung der Virussuppression wirksam erscheinen lassen: Bei keinem Patienten kam es zu sogenannten „viral blips“ (vorübergehend detektierbare Viruskonzentration von 50 – 200 HIV-RNA Kopien/ml Blut), wie sie aus Monotherapie-Studien bekannt sind. Nach einer Beobachtungsphase von 72-96 Wochen zeigten alle Patienten eine anhaltend stabile Virusunterdrückung (< 50 HIV-RNA Kopien/ml Blut).

Die vorliegende Studie soll die Zweierkombination Nevirapin plus Lamivudin in einem multizentrischen Setting prüfen. Die Intervention besteht darin, dass die gut selektionierten Patienten (keine früheren Therapieversager auf NNRTI, schon längere Zeit stabile, gut supprimierende HIV Therapie mit Nevirapin) von ihrer Dreierkombination auf Nevirapin plus Lamivudin umgestellt werden. Die Studie ist als Nicht-Unterlegenheitsstudie im Vergleich mit einer virtuellen Kontrollgruppe (stabile Standardkombinationstherapie mit 0% Therapieversagen) konzipiert. Der primäre Outcome-Parameter, anhand derer die Nicht-Unterlegenheit der Zweierkombination beurteilt wird, ist der Anteil an Patienten mit Therapieversagen gemessen als Viruskonzentration von ≥ 200 HIV-RNA Kopien/ml nach 6, 12, 24, 36 und 48 Therapiewochen. Auch nicht-virologisch begründetes Therapieversagen, Viruskonzentration im Vergleich zu einer historischen Kohorte der SHCS unter einer NVP-enthaltenden Therapie sowie die Häufigkeit von „viral blips“ im Vergleich mit Viruskonzentrationsmessungen in den letzten 2 Jahren vor Einschluss werden als sekundäre Outcome-Parameter ausgewertet. Ebenfalls gemessen wird, ob sich im Verlauf der 48 Wochen das Virusreservoir verändert (Messung der HIV-1 DNA Konzentration) sowie, in einer Subgruppe von Patienten, auch zelluläre Parameter der Immunaktivierung als Marker für aktive virale Replikation.

ABBREVIATIONS

AE	Adverse Event
CA	Competent Authority (e.g. Swissmedic)
CEC	Competent Ethics Committee
CRF	Case Report Form
ClinO	Ordinance on Clinical Trials in Human Research *(in German: KlinV, in French: OClin)*
eCRF	Electronic Case Report Form
cART	Combined antiretroviral therapy
CNS	Central nervous system
CSF	Cerebrospinal Fluid
CTCAE	Common terminology criteria for adverse events
DSUR	Development safety update report
GCP	Good Clinical Practice
IB	Investigator's Brochure
H_o	Null hypothesis
H_1	Alternative hypothesis
HFG	Humanforschungsgesetz (Law on human research)
HMG	Heilmittelgesetz
HRA	Federal Act on Research involving Human Beings
IMP	Investigational Medicinal Product
IIT	Investigator-initiated Trial
ISO	International Organisation for Standardisation
ISF	Investigator Site File
ITT	Intention to treat
KlinV	Verordnung über klinische Versuche in der Humanforschung *(in English: ClinO, in French OClin)*
KSSG	Kantonsspital St. Gallen / Cantonal Hospital St. Gallen
LPTh	Loi sur les produits thérapeutiques
LRH	Loi fédérale relative à la recherche sur l'être humain
MD	Medical Device
OClin	Ordonnance sur les essais cliniques dans le cadre de la recherche sur l'être humain *(in German : KlinV, in English : ClinO)*
PBMC	Peripheral blood mononuclear cell
PI	Principal Investigator
NRTI	Nucleoside Reverse Transcriptase Inhibitor
NNRTI	Non-nucleoside Reverse Transcriptase Inhibitor
SHCS	Swiss HIV Cohort Study
SDV	Source Data Verification
SOP	Standard Operating Procedure
SPC	Summary of product characteristics
SUSAR	Suspected Unexpected Serious Adverse Reaction
TDM	Therapeutic drug monitoring
TMF	Trial Master File

STUDY SCHEDULE

Study Periods	Screening	Observational phase					Follow up
**Visit**	**1**	**2**	**3 - 4**	**5**	**6**	**7**	(if patient prefers to remain on bi-therapy) every 3 months[§]
Time (week)	-4 to 0	0	6, 12	24	36	48	
Time frame +/- weeks			+/- 2	+/-5	+/-5	+6 /-2	
Telephone interview possible[#]	No	No	**Yes**	No	**Yes**	No	
Obtain Informed Consent	x						
Demographics	x						
HIV-treatment History	x						
In- /Exclusion Criteria	x						
Vital Signs[&]	x			x		x	
Laboratory Tests:							
HIV-RNA sampling and analysis[+]		x	x*	x	x*	x	x
HIV-1-DNA (PBMCs sampling)		x				x	
<u>Centre *St. Gallen*</u>:							
Immune activation markers		x				x	
Adherence check[#]	x	x	x	x	x	x	
Concomitant therapy[#]		x	x	x	x	x	
Assessment of SAE[#]		x	x	x	x	x	

* Blood collection for HIV-RNA detection for visit 3, 4 and 6 may be delegated to an external health-care provider.

[#] Telephone interviews instead of visit possible in case blood sampling is performed by an external health-care provider or the patients' general practitioner. In case of occurrence of a SAE that is not clearly unrelated to the study and does not need further evaluation (e.g. hospitalization for elective surgery), the patient will have to come to a clinica assessment at the study site.

[&] Blood pressure, pulse, weight, hip/waist circumference, body temperature.

[§] In patients having >96 weeks of follow-up on NVP + 3TC bi-therapy, follow-up frequency can be reduced to every 6 months if deemed adequate by the treating physician.

[+] Confirmatory visits (within four weeks) are scheduled in case of elevated viral load (HIV-RNA ≥ 50 cp/ml).

1. STUDY ADMINISTRATIVE STRUCTURE

A study personnel (delegation) log in the TMF and the respective ISFs at each site will list all the individuals participating with their responsibilities in the study, including (but not limited to) obtaining (and signing) informed consent, evaluating the patients' HIV treatment history, drug accountability, telephone calls, entering data in the eCRF, etc.

1.1 Sponsor, Sponsor-Investigator

Sponsor:
Cantonal Hospital St. Gallen

Sponsor – Investigator:
Prof. Dr. med. Pietro Vernazza

Division Chief Clinic of Infectious Diseases and Hospital Epidemiology
Cantonal Hospital St. Gallen
Rorschacherstrasse 95
9007 St. Gallen

Phone: +41 71 494 26 31
Mobile: +41 79 666 26 31
Mail: pietro.vernazza@kssg.ch

The sponsor investigator is responsible for the management of the study, data analysis and interpretation of data and writing the final study report. He can delegate some of these tasks to the study coordinator and principal investigator who will support the sponsor investigator, but he still holds the final responsibility for these tasks.

1.2 Principal Investigator(s)

Study Site St. Gallen

Dr. med. Christian Kahlert
Clinic of Infectious Diseases and Hospital Epidemiology
Cantonal Hospital St. Gallen
9007 St. Gallen
Phone: +41 71 494 19 71
Mobile: +41 78 852 02 74
Mail: christian.kahlert@kssg.ch

Study Site Aarau

PD Dr. med. Christoph Fux
Chief Division of Infectious Diseases and Hospital Epidemiology
Cantonal Hospital Aarau
Phone: +41 62 838 68 20
Mobile: +41 79 760 17 17
Mail: christoph.fux@ksa.ch

Study Site Basel

Dr. med. Marcel Stöckle,
Department of Infectious Diseases & Hospital Hygiene
University Hospital Basel
Phone: +41 61 328 66 35
Mobile: +41 78 878 39 57
Mail: marcel.stoeckle@usb.ch

Study Site Zurich

Dr. med. Carsten Depmeier
Arztpraxis Kalkbreite
Badenerstrasse 177
8003 Zürich
Phone: +41 44 350 39 39
Mail: carsten.depmeier@hin.ch

1.3 Statistician ("Biostatistician")

Dr. med. Andrea Bregenzer
Clinic of Infectious Diseases and Hospital Epidemiology
Cantonal Hospital St. Gallen
9007 St. Gallen
Phone: +41 62 838 69 32
Mobile: +41 76 581 2650
Mail: andrea.bregenzer@kssg.ch

1.4 Laboratory

HIV-RNA measurement:
Clinical laboratories of the sites

HIV-1 DNA measurement (week 0, week 48):
Laboratory Dr. K. Metzner, Division of Infectious Diseases and Hospital Epidemiology, University Hospital Zurich

Immune activation markers (week 0, week 48):
KSSG St. Gallen, research laboratory Dr. C. Kahlert

1.5 Monitoring institution

The study monitoring will be performed by the CTU at the KSSG (Director: Dr. Reinhard Maier).

1.6 Data Safety Monitoring Committee

No DSMC planned for this study.

1.7 Any other relevant Committee, Person, Organisation, Institution

The following individuals are members of the protocol development team and/or their role in the study is outlined below:

Pietro Vernazza, Kantonsspital St. Gallen
Protocol Chair, Sponsor investigator.

Christian Kahlert, Kantonsspital St. Gallen
Will serve as local investigator in St. Gallen with responsibility for patient recruitment and follow-up. In addition, he will supervise the analysis of activation markers by flow cytometry in the research laboratory and finally will analyze and report the results. He will support the team writing the first manuscript draft and publishing the study results.

Karin Metzner, Universitätsspital Zürich
Responsible for the quantification of HIV-1 DNA (viral reservoir), analyzing and reporting the HIV-1 DNA data, support in writing and publishing the results

Andrea Bregenzer, Kantonsspital St. Gallen
Protocol statistician (data analysis), primary role in the discussion / development of study design and statistics. Participation in the protocol development team. Collaboration in writing of the final manuscript.

Christoph Fux, Kantonsspital Aarau
Participation in the protocol development team. Will serve as local investigator in Aarau with responsibility for patient recruitment and follow-up. Collaboration writing of the final manuscript.

Marcel Stöckle, Univeritätsspital Basel
Participation in the protocol development team. Will serve as local investigator in Basel with responsibility for patient recruitment and follow-up. Collaboration in writing of the final manuscript.

Carsten Depmeier, Kalkbreite Praxis, Zürich
Participation in the protocol development team. Will serve as local investigator in Zurich with responsibility for patient recruitment and follow up. Collaboration in writing of the final manuscript.

2. ETHICAL AND REGULATORY ASPECTS

The decision of the competent ethics committee (CEC) concerning the conduct of the study will be made in writing to the Sponsor-Investigator before commencement of this study. The clinical study can only begin once approval from all required ethics committees has been received. Any additional requirements imposed by the ethics committees shall be implemented.

2.1 Study registration

The study will be registered in the database ClinicalTrials.gov (listed in the WHO International Clinical Trials Registry Platform (ICTRP, http://www.who.int/ictrp/en/) after receiving approval of the required CEC. In addition, registration in the Swiss Federal Complementary Database (Studienportal SNCTP) will be done in the process of protocol evaluation by the central CEC.

2.2 Categorisation of study

The study is categorized as A due to comprehensive safety data available for the investigational medicinal products both of which have been authorized in Switzerland for HIV therapy for years. According to clinical standards, every HIV therapy is started as a triple drug regimen. Therefore, for all HIV drugs, the summary of product characteristics (SPC) mentions the necessity to start any HIV treatment with a triple drug regimen. This is required in order to prevent resistance in the situation with active viral replication. None of the SPC of any HIV drug covers the questions of simplification therapy for long-term maintenance. However, in recent years, many clinicians have started to reduce the number of drugs in a regimen in order to reduce the risk of adverse events, and this effort to reduce the required exposure to unnecessary drugs has become a standard in many centers. In fact, an editorial comment to the publication of the IAS HIV treatment guidelines 2018 wrote: “Therefore, 2-drug strategies should currently be avoided if possible as initial therapy but appear to be safer for switch therapy” (9). The regimen proposed in this study is not used for another indication, which would qualify for a switch to study category B. The main concern is not the risk of adverse events (since drug exposure is reduced) but the documentation of adequate efficacy. In clinical practice, HIV clinicians are currently exploring many ways to reduce drug exposure (e.g. due to adverse events). The purpose of this clinical study is to systematically evaluate a very attractive option for a HIV dual maintenance therapy and to provide this experience to a larger group of patients.

2.3 Competent Ethics Committee (CEC)

Clinical sites will be in the cantons of Aargau, Basel, Zürich and St. Gallen. The sponsor investigator will make sure that the required local CEC will be contacted for approval and the study will only be started in those centres for which final approval was obtained. Inclusion of additional centres will be reported to the central CEC (“Leitethik-Kommission” = Ethikkommission Ostschweiz).

All changes in the research activity and all unanticipated problems involving risks to humans, or premature termination of the study for any reason will be reported to the ethics committees. No changes are made to the protocol without prior Sponsor and CEC approval, except where necessary to eliminate apparent immediate hazards to study participants.

Premature study end or interruption of the study is reported within 15 days. The regular end of the study is reported to the CEC within 90 days, the final study report shall be submitted within one year after study end. Amendments are reported according to chapter 2.10.

2.4 Competent Authorities (CA)

According to the study category assigned (see 2.2), approval of Swissmedic is not required prior to the start of the clinical trial.

2.5 Ethical Conduct of the Study

The study will be carried out in accordance to the protocol and with principles enunciated in the current version of the Declaration of Helsinki, the guidelines of Good Clinical Practice (GCP) issued by ICH, and the Swiss Law and Swiss regulatory authority’s requirements. The CEC will receive annual safety and interim reports and be informed about study stop/end in agreement with local requirements.

2.6 Declaration of interest

The sponsor and principal investigators confirm that they have no conflict of interest with the manufacturer or distributor of the medicinal products under study.

2.7 Patient Information and Informed Consent

The investigators will explain to each participant the nature of the study, its purpose, the procedures involved, the expected duration, the potential risks and benefits and any discomfort it may entail. Each participant will be

informed that the participation in the study is voluntary and that he/she may withdraw from the study at any time and that withdrawal of consent will not affect his/her subsequent medical assistance and HIV maintenance treatment. All participants will be informed that their study participation can be terminated by the principal investigator in case of questionable adherence of the participant and that this decision will not affect his/her subsequent medical assistance and HIV maintenance treatment.

The participant must be informed that his/her medical records may be examined by authorised individuals other than their treating physician.

All participants for the study will be provided a participant information sheet and a consent form describing the study and providing sufficient information for participant to make an informed decision about their participation in the study. The patient must have enough time to read the patient informed consent form and to discuss the study with his treating physician (written study information should be given at least one day prior to signing the consent form).

The patient information sheet and the consent form will be submitted to the CEC to be reviewed and approved. The formal consent of a participant, using the approved consent form, must be obtained before the participant is submitted to any study procedure.

The participant should read and consider the statement before signing and dating the informed consent form, and should be given a copy of the signed document. The consent form must also be signed and dated by the investigator (or his designee) and it will be retained as part of the study records.

2.8 Participant privacy and confidentiality

The investigator affirms and upholds the principle of the participant's right to privacy and that they shall comply with applicable privacy laws. Especially, anonymity of the participants shall be guaranteed when presenting the data at scientific meetings or publishing them in scientific journals.

Individual subject medical information obtained as a result of this study is considered confidential and disclosure to third parties is prohibited. Subject confidentiality will be further ensured by utilising subject identification code numbers corresponding to treatment data in the computer files.

For data verification purposes, authorised representatives of the Sponsor–Investigator, or an ethics committee or the monitoring institution may require direct access to parts of the medical records relevant to the study, including participants’ medical history.

2.9 Early termination of the study

The Sponsor-Investigator may terminate the study prematurely according to certain circumstances, for example:

- ethical concerns,
- insufficient participant recruitment,
- when the safety of the participants is doubtful or at risk, respectively,
- alterations in accepted clinical practice that make the continuation of a clinical trial unwise,
- early evidence of benefit or harm of the treatment regimen.

Stopping rule in case of true virological failure

The study will be prematurely stopped by the sponsor if three (or more) individual cases with a true virological failure are documented as described in the protocol section 8.6.

2.10 Protocol amendments

Any principal investigator may propose study protocol amendments to the sponsor-investigator. Substantial amendments are only implemented after approval of the CEC.

Under emergency circumstances, deviations from the protocol to protect the rights, safety and well-being of human subjects may proceed without prior approval of the sponsor and the CEC. Such deviations shall be documented and reported to the sponsor and the CEC as soon as possible.

All non-substantial amendments are communicated to the CEC within the Annual Safety Report (ASR).

3. BACKGROUND AND RATIONALE

3.1 Background and Rationale

3.1.1 Standard therapy for HIV

Since 1996, when combined antiretroviral treatment of HIV (cART) with three antiretroviral compounds was established, complete and continued suppression of HIV replication within the body could be achieved. This status of viral control leads to an impressive resolution of most HIV-associated conditions and almost normalizes the immune-activation associated with HIV-infection(10). Once suppression of viral replication is stably reached, no further viral replication and evolution occurs in the body as long as uninterrupted treatment is maintained(11). As a result, life expectancy of HIV infected individuals has almost reached the levels of the general population(12).

Until today, all International guidelines discuss the use of certain antiretroviral combinations to start cART(13). Once treatment is established, HIV infected individuals usually continue with their therapy unless the occurrence of side effects require a change of one or more compounds. Little attention has been given to the distinct features of the initial phase of cART and the long-term maintenance phase of viral suppression, once stable stop of viral replication is reached.

However, there are a few fundamental differences among these two phases: During the initial induction phase, we face a situation of massive viral turnover within the lymphatic tissue. In an average patient with a HIV blood viral load of 4.5 $\log_{10}$ cp/ml, ten billion (10^{10}) new viral particles are created every day(14). As a result of the error prone nature of the viral (reverse) transcriptase (rt) this massive viral replication leads to the occurrence of a large number of mutations(15). On average, during the replication of one viral genome, the viral RT creates 1-3.4 mutations per one hundred thousand base pairs (10^{-5}). The length of the viral genome is 9kbp, or roughly 10^4. Since mutations occur at random, any single basepair of the viral genome can mutate after a single day (16). Thus, main reason to combine three compounds to treat HIV infection is to prevent the escape of drug-resistant mutants. This risk is especially pronounced during the initial phase of therapy when massive viral replication occurs. However, the situation is completely different for the phase of HIV maintenance, i.e. when the viral load has been suppressed and viral replication (and evolution) is completely stopped.

Still, cART with a triple combination has significant drawbacks. Most regimens contain two NRTIs, either abacavir or tenofovir combined with 3TC (lamivudine, or the very similar compound FTC). 3TC has a very long record in HIV-therapy with almost 25 years of clinical experience. It is not only a compound with very limited side effects and can be purchased at lower prices as a generic compound, it also has the advantage of excellent penetration in sanctuary sites. The two other compounds (abacavir or tenofovir) both have the disadvantage of significant long-term side effects. With the recent introduction of a prodrug of tenofovir (tenofovir alafenamide, TAF) some of the side effects of tenofovir are reduced. However, the experience with this young drug does not yet exclude the possibility of long-term side effects (e.g. consequences of the inhibition of telomerase) (17,18).

In the last decade, a number of studies demonstrated excellent results for 80 to 90% of individuals treated during the maintenance phase with PI/r-monotherapy (19) even on the long-term (4). Thus, during the virologically “silent” maintenance phase of HIV-therapy, antiviral suppression appears to be much less demanding. While very strict adherence to cART dosing is crucial for a successful establishment of full viral suppression (initiation phase), patients who fail with their regular drug intake later in therapy (maintenance phase), face much less consequences, i.e. the treatment is much more “forgiving”(20).

3.1.2 Monotherapy in the maintenance phase

Ritonavir-boosted PI-monotherapy was evaluated in a number of studies worldwide. One important observation, quite uniformly shown in all PI/r-monotherapy studies, was the increased likelihood of viral “blips”. Viral “blip” refers to the occurrence of a detectable viral load at levels between 50 and 200 HIV-RNA cp/ml which is usually followed by a level below 50 cp/ml plasma. The consequences of this observation are not yet fully explored, however clinical studies now use the 200 HIV-RNA copy level as the threshold for virological failure (21). The risk of development of a complete viral failure during PI/r-monotherapy was low in most studies, but certainly increased compared with triple cART. Two risk factors for viral failure were established in some studies (not consistently, mostly one or the other factor): A nadir of < 200 CD4 cells/µl was established as the most important risk factor for failure, followed by the (short) duration of cART prior to treatment switch to monotherapy. Due to the clear limitations of monotherapy with a PI/r, this therapy is usually discouraged nowadays by most guidelines.

As a result of these limitations of PI/r-monotherapies, some groups continued to further develop strategies of dual therapies with PIs combined with the nucleoside 3TC (5,6). Although a few studies evaluating this strategy demonstrated an excellent virological suppression with this combination, enthusiasm for this combination was limited due to the significant long-term adverse reaction of ritonavir-boosted PIs, particularly the increase in lipid levels and increased incidence of myocardial infarction (22). However, the long-term efficacy of this combination in HIV maintenance was never challenged.

3.1.3 Monotherapies with dolutegravir (DTG)

With the introduction of the integrase inhibitor DTG, some study groups have started to use DTG as a monotherapy in HIV maintenance. Based on the high genetic barrier (2) and the impressive and rapid suppression of HIV viral load during DTG treatment initiation (3), the expectations were very high for this drug as

monotherapy. Some centres have started to publish retrospective data on DTG monotherapy with mixed results. Observational studies of single arm pilot studies or retrospective analyses for about 24-week duration have been presented. While all these small studies demonstrated continued HIV viral load suppression for up to 24 weeks in the majority of patients, virological failure was nevertheless demonstrated in around 10% of these patients. Despite the initially high expectations for DTG-monotherapy, the unexpected high failure rate in most pilot studies has damped the enthusiasm for "dolu-Mono" among HIV-experts, while the reasons for DTG-mono failures still remains to be investigated.

As was the case for PIs, most groups evaluating drug saving strategies with DTG now use 3TC as an add-on. This is also the case in the Swiss SIMPLE-HIV within the Swiss HIV Cohort which is expected to terminate in 2019. According to our current experience, this regimen remains very effective as was the case for dual therapy with ritonavir boosted PIs and 3TC.

3.1.4 Nevirapine as an underestimated antiretroviral drug

Any reduction in drug exposure for antiretroviral therapy will lower the risk for the development of side effects. In addition, limiting the number of drugs in an antiretroviral combination will also result in reduced cost. Today, the Federal Office of Public Health estimates lifetime costs for cART at about half a Million CHF per infected individual (23). Therefore, a special focus of drug (and therefore cost-) saving strategies should be the evaluation of drugs that are off-patent and could be distributed as generic drugs.

NVP, the first NNRTI that entered the market in the early 90-ies has an excellent record for long-term tolerance and side effects (24). Among all the NNRTIs and PIs, NVP is the only one that has no negative effect on lipids(25). The reason, why NVP is not very frequently used by many physicians is the relatively frequent (15-20%) occurrence of hypersensitivity reactions (either skin rash or elevate liver enzymes) during the first 8-12 weeks of treatment. Since undetected elevation of liver enzymes can result in liver failure during extended treatment with NVP, many physicians did not use this drug, despite its low price.

However, at the HIV clinic at the Kantonsspital St. Gallen we have started in 2003 to use this drug for the antiviral maintenance therapy, after it became apparent that the rate of hypersensitivity reactions was much smaller in patients who have already a suppressed viral load (26,27). As a consequence, NVP is the most frequently used backbone in St. Gallen with more than 220 patients on NVP. Within the SHCS, more than 850 patients are stably treated with a NVP containing regimen.

One outstanding characteristic of NVP is its high penetration into sanctuaries. Among all antiretroviral drugs, NVP is probably the one with the best documented penetration rate in the genital tract and in the CNS(28). Furthermore, it is the only drug for which high levels in brain tissue have also been shown and the drug is also used with excellent results in HIV-encephalopathy / HIV-dementia.

3.1.5 Evaluation of new HIV regimens

The gold standard design of a study evaluating the efficacy of a new drug regimen to treat any medical condition for which a standard therapy is already available is the randomized controlled trial (RCT) comparing the new regimen with the current standard therapy. In situations such as HIV-therapy, where current treatment results are already excellent, a new regimen is usually unable to demonstrate superiority to the standard regimen. As a consequence, a non-inferiority design is chosen to demonstrate adequate efficacy of a new regimen. Non-inferiority trials are intended to show that the effect of a new treatment is not worse than that of an active control by more than a specified margin, the so called non-inferiority margin, which has to be set arbitrarily. In the past, most studies evaluating new regimens in treatment-naïve HIV patients have used a 10 to 12% non-inferiority margin regarding treatment success (HIV-RNA <50 cp/ml). In previous treatment switch trials, the rate of virological failure (proportion of patient's ≥50 cp/mL) was typically in the range of 1-3%. Assuming 2% will have HIV-RNA ≥50 cp/mL at 48 weeks in both treatment arms, the US regulatory agency (FDA) considers a non-inferiority margin of 4% for virological failure in switch trials to be clinically tolerable(29).

Overall, patients (n=9116) in the SHCS reach a HIV-suppression rate (<200 RNA cp/ml) of 98.2%. In patients on NVP, the suppression maintenance rate is even higher (99.2%). Among the 250 NVP-treated patients in St. Gallen and Aarau we identified only 6 patients with a viral load value above 50 HIV-RNA cp/ml. Treatment failure was clearly associated with either a treatment holiday for personal reasons or a psychiatric illness. One of the six patients had a viral blip (160 HIV-RNA cp/ml) that was resolved at the next viral load measurement (i.e. not qualifying as failure). In summary, patients with reliable drug adherence consistently maintain their virologic suppression.

With this high efficacy of HIV maintenance therapy, we have to question the role of a comparison group in the RCT. If patients who stop antiretroviral therapy on purpose or who have a significant psychiatric comorbidity are excluded, the suppression rate after one year in the control arm in a RCT reaches 100%. Assuming a 100% suppression rate in the standard control arm would thus only **under**estimate the efficacy of the investigational therapy but certainly not overestimate it.

In this study, we therefore also propose to use a non-conventional, innovative approach to evaluate a new maintenance strategy by evaluating non-inferiority of the new regimen compared to the virtual comparison arm for which 100% success rate (or 0% virological failure rate) is assumed. This design results in a significant reduction of study complexity, sample size and cost.

The proposed novel study design was discussed (among others) with an expert in clinical study design at the

CTU of the University Bern (personal communication Sven Trelle) and he supported the proposed study design of a non-inferiority design comparing the new intervention with a virtual control group for which an 100% success rate would be assumed(30).

3.1.6 Own Experience in the Field

The ID clinic in St. Gallen was the first clinic that investigated the use of ritonavir boosted PI-monotherapy almost 15 years ago(31) in a small study (n=12). The study was the first to demonstrate excellent 48-week suppression during maintenance, but it already demonstrated the increased rate of viral blips and also an inacceptable high rate of side effects under ritonavir boosted indinavir (Figure 2). At that time, we already had provided some evidence for a compartmentalization of the genital tract regarding penetration of PIs(32). More evidence for compartmentalization was found both for the genital tract as well as for the central nervous system by other investigators. This led us to suspect that the poor penetration of PIs into the compartment could be a reason for the unexplained but consistent observation of viral blips during monotherapy.

As a consequence, the next study – this time including other centers of the SHCS to include a total of 30 patients - was evaluating the effect of ritonavir boosted atazanavir monotherapy on the sanctuary sites genital tract and CNS(33). This study was the first to demonstrate the limited activity of ritonavir boosted PIs in the CNS when given as monotherapy. The next step was conducted in the "MOST" study with all SHCS centers, where the CNS penetration was evaluated in a large randomized study with 100 patients on lopinavir-monotherapy (SNF-Project 32473B-114006). However, that study had to be terminated early due to the demonstration of unacceptably high viral breakthrough in the CSF in patients randomized into the monotherapy arm(34).

Fig 1: Activation markers in Lopinavir MOST-Study

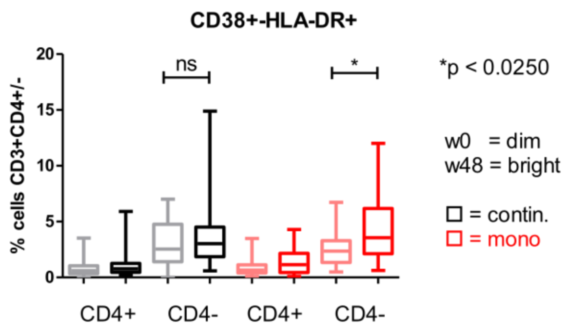

In the randomized MOST study, we also studied immune activation as a possible marker for active viral replication. Comparison of the best characterized activation markers CD38+ and HLA-DR+ on CD4 negative T-lymphocytes showed a significant difference in patients switched to monotherapy (Figure 1) while immune activation was not enhanced in patients on continued triple therapy (see Figure, data not published). This observation may in fact indicate ongoing replication in the sanctuaries. Some patients participating in these studies continued monotherapy after completion of the study. A recent analysis of the long-term experience in patients on monotherapy revealed late treatment failures, particularly in the CNS, in 1/3 of these patients(4).

A small pilot study in 20 patients with the combination proposed here (NVP+3TC) was conducted in St. Gallen. After more than 1300 weeks of observation, none of these 20 patients has failed this NVP/3TC dual therapy(35). More importantly, not a single case with a viral blip was observed in this pilot study. Figure 2 demonstrates the comparison of blips in this pilot (right) with the blips observed in our first PI/r monotherapy with indinavir(31).

Fig. 2: Viral "blip" analysis in Indinavir (PI/r) Mono study and NVP+3TC Pilot study

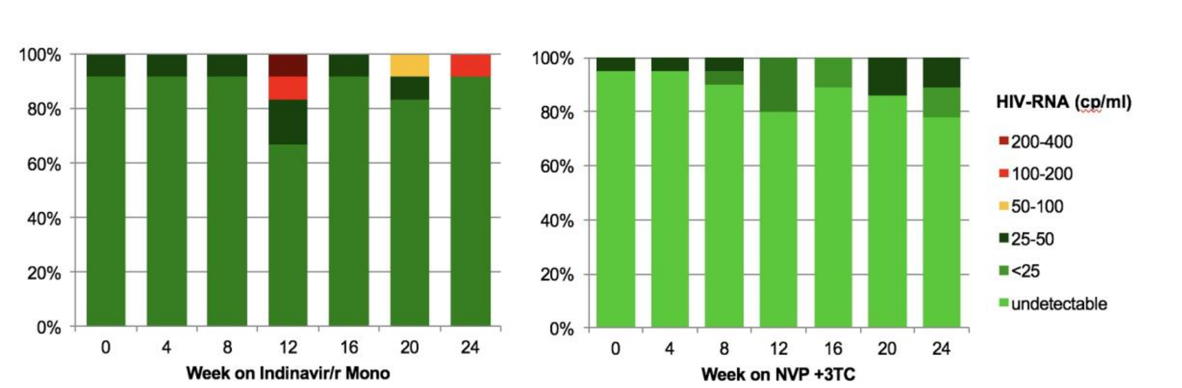

The lack of any detectable increase in the frequency of viral blips in the NVP + 3TC pilot suggests the absence of residual viral replication in this regimen which was the supposed mechanism for the observed blips in the PI/r-monotherapy studies.

As discussed above, PIs have a weaker penetration into the CSF while NVP and 3TC belong to the drugs with the best penetration into the CSF and other sanctuary sites, such as the genital tract. The somehow unexpected high efficacy of NVP/3TC in our pilot study lets us hypothesize that compartment penetration rather than barrier to resistance is the main reason for failure in HIV maintenance therapy.

We therefore aim to evaluate in this properly powered study the efficacy of NVP + 3TC dual therapy to maintain virologic suppression in patients on a NVP containing HIV regimen to support of our hypothesis.

3.2 Investigational Product (treatment, device) and Indication

As described in 3.1, the study examines the efficacy of two instead of three standard antiretroviral drugs in HIV maintenance therapy. Both drugs have been well-established as single agent preparations and (for lamivudine) in fixed-combination preparations in cART since more than 25 years.

3.3 Preclinical Evidence

Not applicable.

3.4 Clinical Evidence to Date

As described in 3.2.6, a pilot study with 20 treatment-experienced HIV-1 patients at the Clinic of Infectious Diseases in St. Gallen shows excellent results (35). The study demonstrates treatment success (no virological failure and no viral blips) in all patients switching to NVP/3TC bi-therapy after more than 1300 observation weeks (data not published).

3.5 Dose Rationale / Medical Device: Rationale for the intended purpose in study (pre-market MD)

Dosage and dosage regimens of NVP and lamivudine are as described in the respective SPC (standard dosing: Nevirapine 400mg retard tablets once daily, lamivudine 300mg tablets once daily).

3.6 Explanation for choice of comparator (or placebo)

The rationale behind a virtual control group representing the assumption of 0% failure rate or 100% viral suppression rate in patients on maintenance therapy (i.e. the optimum) is that in a non-inferiority trial the effect of the investigational therapy would be underestimated at its best, but certainly not overestimated (as described in 3.1.5).

If patients who stop antiretroviral therapy on purpose or who have a significant psychiatric comorbidity are excluded from the study, a viral suppression rate of 100% is highly likely to be achieved, and may therefore be postulated for the control arm.

3.7 Risks / Benefits

The main concern of a reduction of active compounds in an antiretroviral combination therapy is the potential loss of antiretroviral activity. As discussed in section 3.1.1, the risk of treatment failure is much lower in the situation of maintenance therapy, when the patients' viral load is continuously suppressed. Loss of activity of a treatment requires the development of viral mutations associated with resistance. However, in order to develop a mutation, the virus must replicate in vivo (in the presence of selection pressure).

Despite the theoretical considerations and the good experience in the pilot study mentioned above, development of resistance remains a concern. The following strategies to minimize the risk are implemented:

- Frequent HIV-RNA testing (every 6 weeks up to week 12, then every 12 weeks) in order to rapidly detect every slightest increase in viral replication.
- Implementation of a stopping rule to prevent the occurrence of more than three true virological failures (≥200 HIV-RNA cp/ml confirmed by a 2nd HIV-RNA measurement within 4 weeks) with consecutive development of resistance. If three or more patients demonstrate true virological failure during the study, the study will be terminated prematurely (see section 8.6). However, patients, who plan to stop participation in the study or antiretroviral therapy will not be considered as patients failing treatment, as long as their HIV-RNA measurement at the time of the decision still demonstrated virological suppression. The data acquired up to the treatment interruption will be used for further analysis.

In order to minimize the chance to inadequately classify patients as virological failures, patients will be informed that they may terminate the study at any time point, but:

- Patients will be asked to have a viral load-measurement prior to terminating the intervention treatment in order to demonstrate full suppression.
- Patients who decide for a treatment termination will remain in the analysis

Possible adverse events:

The occurrence of drug adverse events is always possible during any therapy. However, the risk having a side effect is definitively lower in patients who take only two drugs instead of three. All study patients will have been on a stable NVP containing regimen at study initiation for more than 6 months. Therefore, there is no risk of hypersensitivity reactions to NVP, which by definition occurs within the first 3 months of therapy.

3.8 Justification of choice of study population

The study protocol proposes to include treatment-experienced HIV patients on a stable NVP-based treatment for at least 6 months and viral load suppression for more than 2 years prior to inclusion. At the HIV centre in St. Gallen, this is the largest patient group among all drug regimens used. The patients need to be on a NVP-based regimen to prevent the occurrence of hypersensitivity reactions during the study.

4. STUDY OBJECTIVES

4.1 Overall Objective

The purpose of the study is to evaluate non-inferiority of nevirapine/lamivudine bi-therapy compared to an optimal (virtual) HIV maintenance therapy defined. The advantages of the proposed regimen are its good long-term tolerability and low cost (generic drugs).

4.2 Primary Objective

The primary objective of the trial is to determine if the virological failure rate during 48 weeks on a NVP+3TC bi-therapy is non-inferior to an (idealized) optimal standard therapy in patients fulfilling the entry criteria.

Entry criteria (see 7.1) include a stable HIV therapy for 24 months with at least 6 months on a nevirapine containing regimen for at least 6 months prior to inclusion. For definition of non-inferiority see section 11.2. For definitions of virological failure see section 5.2

4.3 Secondary Objectives

The secondary objective of the study is to detect any other indication for a reduced antiretroviral activity of the bi-therapy. This includes the evaluation of

- residual viral replication indicated by
    - Increase in frequency of viral blips
    - Increase in the HIV reservoir size
    - Increase in immune activation markers (such as CD8+ and CD4+ T-cells)

4.4 Safety Objectives

The treatment regimen under study consists of two drugs well-known in HIV therapy. Both NVP and 3TC have shown excellent long-term tolerability and, moreover, in a pilot study have proved effective by demonstrating no evidence of virological failure. The latter will be the primary endpoint of this study, and along with the implementation of a regular HIV-RNA blood monitoring and the investigators' experiences with switching patients on NVP, the proposed treatment regimen has a limited risk of patient safety issues. Since antiretroviral therapy is generally seen as an advantage to any HIV-infected patient and all patients have been on an antiretroviral therapy for a minimum of two years prior to this study, reducing the number of active drugs can only result – if anything – in a reduction of the risk of drug associated adverse events.

Therefore, safety objectives are restricted to recording serious adverse events (SAE) and suspected unexpected serious adverse reactions (SUSAR, see also 10.).

5. STUDY OUTCOMES

5.1 Primary Outcome

As the primary efficacy endpoint for treatment switch trials, the FDA recommends the proportion of patients with HIV-RNA greater than or equal to a certain level at 48 weeks. This endpoint focuses on virological failure and not success. This is because in switch trials patients are starting with HIV-RNA levels that are already suppressed below the assay limit of quantification. In other words, the endpoint of interest is the proportion of people with suppressed HIV-RNA at baseline who lose virological control after switching to a new drug or regimen (36, FDA Guidance, p.21).

Previous switch trials have used the proportion of patients with HIV-RNA below 50 cp/ml as the primary endpoint. However, most of the patients who were not counted as treatment successes did not experience actual virological failure but were not considered successes for other reasons, such as stopping the drug because of adverse reactions, lost to follow-up, or withdrawing consent, among others (36, page 21).

In this study, the primary outcome is the proportion of patients with HIV-RNA ≥ 200 cp/ml at week 48 (confirmed by a second measurement within 4 weeks) (36, FDA Guidance, snapshot approach, described in Appendix A, pp 33-36).

Further details on the analysis plan are given in section 11.4.2.

5.2 Secondary Outcomes

Secondary endpoints of the study are aiming at detection of alternative evidence of reduced antiretroviral activity of the regimen.

Signs of potentially reduced antiretroviral activity are:

a) Increase in frequency of low level HIV-RNA detection
b) Increase in HIV reservoir size
c) Increase in immune activation markers (such as CD8+ and CD4+ T-cells)

Categorization of HIV-RNA measurements:

HIV-RNA is measured in plasma. For the purpose of detection of low level HIV-RNA replication, the following categories based on viral load assessment are defined:

A) Full viral load suppression
   - Viral load suppression is defined as sustained HIV-RNA measurements of < 50 cp/ml

B) Viral blips
   - Viral blips are HIV-RNA measurements ≥ 50 and < 200 cp/ml confirmed by a second measurement < 50 cp/ml within 4 weeks
     Patients who do not show up for the confirmatory measurement within 4 weeks are categorized as *low-level replication*.

C) Low-level replication
   - is defined as repeated HIV-RNA measurements ≥ 50 and < 200 cp/ml
   - is further categorized in 20-49, 50-99 and 100-199 cp/ml for the purpose of the analysis

D) Virological failure
   - is defined as an HIV-RNA measurement of ≥ 200 cp/ml confirmed by a second value ≥ 200 cp/ml within 4 weeks
   - Patients who do not show up for the confirmatory measurement within 4 weeks are considered as virological failure.

5.3 Other Outcomes of Interest

None.

5.4 Safety Outcomes

See section 4.4

6. STUDY DESIGN

6.1 General study design and justification of design

The present protocol describes a comparative open-label non-inferiority multicentre intervention study evaluating virological failure of the intervention arm compared with a virtual control arm for which a failure rate of 0% is assumed. The study is using a 4% non-inferiority margin. The non-inferiority (NI) margin is the pre-specified maximum acceptable extent by which a treatment is less efficacious than an active control. Hence, a non-inferiority trail is intended to show that the effect of a new treatment is not worse than that of the active control by more than this margin. With continuous improvements and already excellent clinical outcomes in HIV therapy, the benefits of a new treatment regimen may be only marginal over existing treatments and difficult to show. In such circumstances, the use of a non-inferiority design is preferred over a superiority trial to demonstrate adequate efficacy.

The intervention arm is the combination of NVP (400mg retard) and 3TC (300mg) in 200 HIV-positive patients who have been receiving cART including nevirapine for at least 6 months. The comparator, i.e. a virtual comparison group, represents the optimal HIV maintenance treatment with 0% treatment failure, which in a non-inferiority design may result in underestimation of the treatment's efficacy at the most, but not in overestimation. The choice of a virtual control arm with 100% viral suppression presumes that treatment adherence remains fully maintained. Thus, patients who stop cART deliberately will be excluded from this study as long as they announce the treatment interruption before their last viral load measurement documenting virological suppression.

The study observation period will be 48 weeks including visits for blood draws and HIV-RNA detection at screening/baseline, week 6, 12, 24, 36 and 48. Participants may visit their general practitioner for the blood draws, but are required to be clinically followed by their treating physician (local investigator) every six months (week 24 and 48).

6.2 Methods of minimising bias

6.2.1 Randomisation

Not applicable.

6.2.2 Blinding procedures

Not applicable.

6.2.3 Other methods of minimising bias

Not applicable.

6.3 Unblinding Procedures (Code break)

Not applicable.

7. STUDY POPULATION

7.1 Eligibility criteria

Participants fulfilling all of the following <u>inclusion</u> criteria are eligible for the study:

- >18 Years old
- Patients on a stable HIV-therapy containing NVP for at least six months
- Duration of viral load suppression (HIV-RNA <50 copies/ml) of more than 2 years (allowing occasional blips, i.e. viral load measurements of 50-200 HIV-RNA cp/ml followed by an additional measurement of <50 cp/ml). Viral load suppression must be documented with at least one HIV-RNA measurement every six months during the 2 years prior to inclusion.
- No previous failure of any NNRTI based therapy
- No known resistance to NVP or 3TC
- Able to fully understand the informed consent and the experimental nature of the study
- Absence of any health believe systems that might interfere with drug intake

The presence of any one of the following <u>exclusion</u> criteria will lead to exclusion of the participant:

- Chronic hepatitis B infection (HBs-AG positive)
- Women who are pregnant or breast feeding,
- Women with childbearing potential who are not willing to use a safe contraception such as an intrauterine device
- Any condition, that might, at the discretion of the principle investigator, interfere with treatment adherence or regular HIV-RNA testing appointments, such as
  i) psychiatric disorders,
  ii) known adherence problems in the past 2 years or
  iii) health believe issues known to cause patients to stop treatment (such as expecting God to cure HIV or not believing the existence of HIV, etc.)

7.2 Recruitment and screening

Study participants are recruited from the pool of patients of out-patient clinics of regional hospitals, of an University hospital and a private practice collaborating in the SHCS and experienced with the care and treatment monitoring of HIV infected patients. Before inclusion, viral load of eligible patients will be measured (see also 9.2 and 9.3., Study Assessments).

If financial support allows, patients may be compensated for travel expenses up to a maximum of 50 CHF per visit.

7.3 Assignment to study groups

Not applicable.

7.4 Criteria for withdrawal / discontinuation of participants

According to the snapshot analysis: see sections 5.1 and 11.4.2 to 11.4.5

For follow-up of withdrawing participants, participants who discontinued their treatment or participants with a severe safety issue, see descriptions in sections 8.4, 9.2.5, 10.1.1

8. STUDY INTERVENTION

8.1 Identity of Investigational Products (treatment / medical device)

The experimental intervention is the treatment with single agent preparations of nevirapine and lamivudine that have been established in cART since more than 25 years. Standard dosages of both nevirapine (400mg retard once daily) and lamivudine (300mg once daily) will be used.

8.1.1 Experimental Intervention (treatment / medical device)

Any generic formulation of drug currently on the Swiss market (Viramune®, Nevirapin Mylan®, Nevirapin-Mepha® Retard, Nevirapin Sandoz® Retard) can be used according to the local preferences or drug availability. The respective brand names are to be recorded in the eCRF.

8.1.2 Control Intervention (standard/routine/comparator treatment / medical device)

Not applicable (see also 3.6 Explanation for choice of comparator).

8.1.3 Packaging, Labelling and Supply (re-supply)

Commercially available products are used. No study specific labelling is required. Drugs are charged to the health care insurance as for standard therapy.

8.1.4 Storage Conditions

The preparations that are used in this study will be stored as recommended in the SPC.

8.2 Administration of experimental and control interventions

8.2.1 Experimental Intervention

The experimental intervention consists of the treatment with two marketed single preparations: nevirapine (400mg) and lamivudine (300mg), both taken orally once daily for the whole duration of the study. All patients must have been already on a NVP-containing regimen for at least 6 months at study initiation.

After having reached the end of the observation period of the study at week 48, successfully suppressed patients may decide to prolong the treatment beyond 48 weeks and the continued observation of these patients is conducted in the framework of the Swiss HIV Cohort Study (SHCS). Continuous monitoring of viral load is suggested every 3 months after the study termination. The frequency of the measurement might be reduced during after an extended observation period of 96 weeks according to the discretion of the treating physician.

8.2.2 Control Intervention

Not applicable.

8.3 Dose modifications

No dose adjustments are planned for this study. If a participant shows virological failure, maintenance therapy with the investigational treatment regime will be discontinued and the participant will be switched to an adequate treatment regimen (see also 7.4 Criteria for withdrawal/discontinuation of participants).

8.4 Compliance with study intervention

Inclusion/exclusion criteria are defined such that mainly patients with adequate adherence are included. Furthermore, the number of tablets per day will be the same as with the treatment before switching treatment At each visit, patients are asked whether they remember having missed a treatment dose, how often they have missed more than one consecutive dose or if they have encountered any other problem that might interfere with optimal adherence.

Therapeutic drug monitoring (TDM): At visits for confirmation of increased viral load (≥ 50 cp/ml), an additional plasma sample is stored for potential TDM. When and if TDM is performed in frozen plasma samples, will be decided by the protocol team on a case-by-case basis when the RNA measurements give reason to questionable adherence.

8.5 Data Collection and Follow-up for withdrawn participants

If participants discontinue treatment for any reason, they will still remain in the study. Their data will be used for the analysis of outcomes as described in section 11.4.5. Patients who withdraw their consent will be asked if the information collected up to the discontinuation time-point may still be used for the study and the data will be used accordingly.

Data for withdrawn subjects will be entered into the patient chart as well as into the electronic case report forms (eCRF).

8.6 Trial specific preventive measures

Any patient failing the experimental treatment, i.e. HIV-RNA ≥ 200 cp/ml confirmed by a second measurement within 4 weeks, will be informed by phone immediately (within 48 hours) by the principle investigator to make an appointment and to define an adequate rescue treatment, which will be started immediately and recorded in the eCRFs.

In order to prevent the occurrence of more than three true virological failures, all events of a viral load measurement ≥ 200 cp/ml will have to be reported immediately (within 48 hours) to the sponsor. The sponsor will inform all site investigators of the occurrence of a virological failure and the results of the further analysis. In failing patients, a detailed treatment history is taken and HIV-resistance testing is performed.

True virological failure will be considered if the patient did not completely interrupt his therapy and resistance testing demonstrates the occurrence of mutations associated with NVP or 3TC resistance. If three true virological failures are detected, the trial will be prematurely stopped, in order to prevent further resistance development.

If three virological failures do occur without fulfilling the criteria of true virological failure, the sponsor will discuss with the protocol team the nature of the observed failing patients and the protocol team will decide within 2 weeks how to continue the trial. All the ethical review boards of the participating centres will be informed about this decision within 15 days.

8.7 Concomitant Interventions (treatments)

Any concomitant drug therapy and changes of concomitant medication should be recorded in the e-CRF during the study. It is in the responsibility of the treating physician to check for potential drug interactions using online resources such as HIV-druginteractions.org or ePocrates.com.

Concomitant treatment interventions will be recorded at each visit according to the standard procedures of clinical patient observation.

8.8 Study Drug Accountability

The patient will receive their study drug via the usual way, i.e. either through the pharmacy or directly dispensed at the treatment centre. The drugs are regularly charged by the health insurance.

8.9 Return or Destruction of Study Drug

Not applicable.

9. STUDY ASSESSMENTS

9.1 Table of study procedures and assessments

**Study Periods**	**Screening**	**Observational phase**					**Follow up**
**Visit**	**1**	**2**	**3 - 4**	**5**	**6**	**7**	(if patient prefers to remain on bi-therapy) every 3 months[§]
Time (week)	-4 to 0	0	6, 12	24	36	48	
Time frame +/- weeks			+/- 2	+/-5	+/-5	+6 /-2	
Telephone interview possible[#]	No	No	**Yes**	No	**Yes**	No	
Obtain Informed Consent	x						
Demographics	x						
HIV-treatment History	x						
In- /Exclusion Criteria	x						
Vital Signs[&]	x			x		x	
Laboratory Tests:							
HIV-RNA sampling and analysis[+]		x	x*	x	x*	x	x
HIV-1-DNA (PBMCs sampling)		x				x	
<u>Centre *St. Gallen*:</u>							
Immune activation markers		x				x	
Adherence check[#]	x	x	x	x	x	x	
Concomitant therapy[#]		x	x	x	x	x	
Assessment of SAE[#]		x	x	x	x	x	

* Blood collection for HIV-RNA detection for visit 3, 4 and 6 may be delegated to an external health-care provider.

[#] Telephone interviews instead of visit possible in case blood sampling is performed by an external health-care provider or the patients' general practitioner. In case of occurrence of a SAE that is not clearly unrelated to the study and does not need further evaluation (e.g. hospitalization for elective surgery), the patient will have to come to a clinica assessment at the study site.

[&] Blood pressure, pulse, weight, hip/waist circumference, body temperature.

[§] In patients having >96 weeks of follow-up on NVP + 3TC bi-therapy, follow-up frequency can be reduced to every 6 months if deemed adequate by the treating physician.

[+] Confirmatory visits (within four weeks) are scheduled in case of elevated viral load (HIV-RNA ≥ 50 cp/ml).

9.2 Assessments of outcomes

9.2.1 Assessment of primary outcome

HIV-RNA measurement is a routine diagnostic parameter and is done by quantitative PCR (qPCR) in plasma in the routine clinical laboratories of each study site. Quantitative HIV-RNA measurement is performed at each visit. In patients living remotely from the study centre, visits 3,4 and 6 can be done by telephone by a trained co-investigator and the blood draw (for HIV-RNA) can be delegated to the general practitioner or another health care provider closer to the patient. Samples drawn by the participants' general practitioner will be sent to the same clinical laboratories.

Results of viral load measurements will be checked by the local principal investigator.

In the case of an HIV-RNA detection ≥ 50 cp/ml, the patient will be called for a second confirmatory viral load measurement that will be scheduled within 4 weeks of the preceding measurement (i.e. the increased value).

The primary endpoint, i.e. virological failure, is reached if a patient has an HIV-RNA ≥ 200 cp/ml with a confirmatory result within 4 weeks.

If the confirmatory blood sample results in an HIV-RNA value below 50 cp/ml, the patient will be followed according to the planned schedule.

If the confirmatory HIV-RNA measurement is within the range of 50-200 cp/ml (i.e. primary endpoint of viral failure not reached), another plasma sample will have to be obtained within 4 weeks.

Assessment of reasons for virological failure or treatment interruption:

- In every patient who experiences a virological failure (HIV-RNA ≥ 200 cp/ml), the treating physician or principal investigator will have to evaluate the possible reasons for the reduced antiviral activity. A thorough medical history will be obtained to evaluate any problem in drug adherence or intentional treatment interruption.
- At the time of the confirmatory blood draw for virological failure, an additional plasma sample is taken and stored at -80°C for potential HIV resistance testing (in the standard laboratory of the participating centre). Resistance testing is performed in case the confirmatory blood draw confirms virological failure (HIV-RNA ≥ 200 cp/ml)

9.2.2 Assessment of secondary outcomes

Alternative indirect evidence for reduced antiviral activity will be evaluated with three approaches:

- *Analysis of blip frequency/low level replication:*
  The frequency of viral blips (3 categories, including 20-49, 50-99, and 100-199 cp/ml) over the 48 week observation period will be compared with two historical controls:
  1) frequency of blips (by category) during bi-therapy with the frequency prior to the study in the same patient
  2) frequency of blips (by category) compared to other SHCS participants treated in the participating centres with NVP

- *Analysis of HIV reservoir size:*
  The size of the HIV-reservoir is estimated by HIV-DNA detection as outlined below. The mean change of HIV-DNA from baseline to week 48 is calculated. An absolute increase of HIV-DNA above the variability of the method would be considered as an indirect indication for an increased HIV reservoir.

  HIV-1 DNA levels in PMBCs at baseline and 48 weeks of treatment will be measured using a droplet digital PCR (ddPCR) system (RainDrop, RainDance Technologies). The samples will be stored at the laboratories associated to the study sites following the SHCS biobank protocol for freezing living cells and sent in one batch to the participating laboratory (K. Metzner, see below).

- *Immune activation marker (study centre St. Gallen):*
  Immune activation markers on CD8 cells (CD38+ and HLA-DR+) are measured by flow cytometry in patients of the site in St. Gallen. A positive mean change in the frequency of activated CD8 cells from baseline to week 48 would serve as a potential indication for an increased low-level viral replication. The flow cytometry is performed according to standard protocols on fresh heparinized blood samples in the research laboratory of C. Kahlert at the Kantonsspital St. Gallen (Visit 2 and 7).

Detailed methodology for the determination of cellular HIV-DNA (“HIV-reservoir”)

The PBMC samples are collected at visit 2 and 7, sent to the site-associated laboratories, frozen according to the SHCS biobank protocol for freezing living cells and stored at least at -80°C to ship them in one batch to the laboratory of K. Metzner (Division of Infectious Diseases and Hospital Epidemiology, University Hospital Zurich).

Peripheral blood mononuclear cells (PBMCs, $3x10^6$ cells per sample) are collected at week 0 and week 48 and frozen according to the SHCS biobank protocol for freezing living cells by the associated laboratories which also handle the vital cell repository within the SHCS. Cells will be stored at least at -80°C until the end of the study and shipped in one batch including both samples from all patients to the participating laboratory (K. Metzner, University Hospital Zurich, Division of Infectious Diseases and Hospital Epidemiology).

The quantification of total cellular HIV-1 DNA will be performed using a novel digital droplet PCR-based assay developed by K. Metzner for the HIVX project “Deciphering Host-Virus Interactions to Cure HIV” (PI Huldrych Günthard, Co-investigator K. Metzner among others). Briefly, DNA will be isolated from 3 million PBMCs, fragmentated, and digital droplet PCR (ddPCR) will be performed using the RainDrop ddPCR system from Raindance. In millions of picoliter-sized droplets, single copy PCRs will be carried out amplifying HIV-1 DNA and the single copy gene C-C chemokine receptor type 5 (CCR5), the latter to determine the input cell number. Within the HIVX mentioned above, this method has been successfully applied to more than 3’500 patients’ cell samples so far. HIV-1 DNA levels will be reported as absolute HIV-1 DNA copy number per one million genomic equivalents.

Data and results of laboratory tests will be entered into the clinical database (secuTrial®) at the respective study centre, and the laboratory document will be stored for 10 years.

Results from the two research laboratories (HIV-reservoir, immune activation) will be provided in a separate document for each study centre. All the procedures and results will be summarized in the report. The report will be kept with the TMF and the respective ISFs stored for 10 years.

9.2.3 Assessment of other outcomes of interest

Any assessment that is part of a routine clinical care in HIV-positive patients will have to be conducted according

to standard procedures, e.g. DEXA-scan in patients with osteopenia or six-monthly CD4/CD8 determination as part of the SHCS-follow-up. However, these additional procedures are not part of the current study evaluation.

9.2.4 Assessment of safety outcomes

9.2.4.1 Adverse events

Recording of adverse event information is restricted to serious adverse events (SAE) and suspected unexpected serious adverse reactions (SUSAR, see also 10 for definition, reporting responsibilities and reporting timelines).

Any spontaneous report will be collected. Study patients are requested to contact their study investigator or, in case of urgency, the sponsor investigator to report serious adverse events. Telephone numbers of study investigators and sponsor investigator are provided at the beginning of treatment. The study investigator will document all SAEs and SUSARs in the clinical chart and eCRF, and report them to the sponsor and ethics committee using a reporting form provided by the CTU St. Gallen. Patients will be asked specifically about serious adverse events at every follow-up visit.

Patient who report a potential side effect or SAE during the telephone interview (visit 3, 4, 6) will be asked to come to the centre for further analysis of the event, if the event requires more detailed information.

9.2.4.2 Laboratory parameters

Not applicable.

9.2.4.3 Vital signs

Height is measured at baseline. Vital signs (blood pressure, pulse, weight, hip/waist circumference, body temperature) will be assessed at baseline, visit 5 (24 weeks) and visit 7 (48 weeks) according to the routine procedure standardized in the SHCS.

9.2.5 Assessments in participants who prematurely stop the study

See also sections 8.6. and 10.1.1. and 10.1.3

Subjects who withdraw from further participation in the study will be asked whether the data collected so far can be used for the analyses. If the participant agrees, data will be used for the analysis of outcomes up to the week of withdrawal.

9.3 Procedures at each visit

9.3.1 Visit 1 / Screening visit (week -4 to 0)

- Informing patient about the background and all details of the study, which may also occur during a routine visit of the patient (the written study information should be given at least one day prior to signing the consent form)
- Obtaining informed consent
- Collecting demographic data (gender, age)
- Collecting HIV-treatment history information
- Verifying inclusion and exclusion criteria
- Checking vital signs (height, weight, hip/waist circumference, blood pressure, pulse, body temperature)
- Scheduling of next visit

As long as the patient has received and read the patient information at least 24 hours prior to the screening visit, the baseline visit can be performed at the same time as the screening visit.

9.3.2 Visit 2, Baseline (week -4 to 0, sampling for HIV-1 DNA quantification and immune activation markers)

- Blood withdrawal for baseline viral load measurement (HIV-RNA)*
- Blood withdrawal for baseline HIV-1-DNA measurement in PBCMs
- Blood withdrawal (heparinized fresh blood) for immune activation markers (St. Gallen patients only)
- Checking vital signs (height, weight, hip/waist circumference, blood pressure, pulse, body temperature)
- Recording of any concomitant medications
- Adherence check, occurrence of any events interfering with patient's adherence
- Scheduling of next visit

*Patients who have an HIV-RNA measurement at the baseline ≥50 cp/ml will be excluded a posteriori from the participation in the study.

9.3.3 Visit 3 (± 2 weeks), 4 (± 2 weeks), and 6 (± 5 weeks), (sampling for HIV-RNA quantification)

- Blood withdrawal for viral load measurement (HIV-RNA)
- Recording of any concomitant medications
- Recording of any concomitant medications
- Recording of SAE

- Adherence check, occurrence of any events interfering with patient's adherence
- Scheduling of next visit

Note: Interview can be done by telephone if blood draw (HIV-RNA) is delegated to another health care provider

9.3.4 Visit 5 (week 24 ± 5 weeks; sampling for HIV-RNA quantification and clinical follow-up)

- Blood withdrawal for viral load measurement (HIV-RNA)
- Checking vital signs (weight, hip/waist circumference, blood pressure, pulse, body temperature)
- Recording of any concomitant medications
- Recording of SAE
- Adherence check, occurrence of any events interfering with patient's adherence
- Scheduling of next visit

9.3.5 Visit 7 (week 48 +6/-2 weeks; sampling for quantification of HIV-RNA-, HIV-1 DNA- and immune activation markers and clinical follow-up)

- Blood withdrawal for viral load measurement (HIV-RNA)
- Blood withdrawal for HIV-1-DNA measurement in PBCMs
- Blood withdrawal (heparinized fresh blood) for immune activation markers (St. Gallen patients only)
- Checking vital signs (weight, hip/waist circumference, blood pressure, pulse, body temperature)
- Recording of any concomitant medications
- Recording of SAE
- Adherence check, occurrence of any events interfering with patient's adherence
- Scheduling of next visit

9.3.6 Confirmatory visit

Additional visits within four weeks) are scheduled in case of elevated viral load (HIV-RNA ≥50 cp/ml).

- Blood withdrawal for viral load measurement (HIV-RNA)
- Asservation of blood sample for drug resistance testing
- Asservation of plasma sample for eventual TDM (therapeutic drug monitoring) if deemed necessary by the protocol team at the end of the study
- Documentation of date and time of last drug intake and blood withdrawal (TDM)

10. SAFETY

10.1 Drug studies

During the entire duration of the study, all serious adverse events (SAEs) are collected, fully investigated and documented in source documents (patient file) and case report forms (eCRF). Study duration encompassed the time from when the participant signs the informed consent until the last protocol-specific procedure has been completed, including a safety follow-up period.

10.1.1 Definition and assessment of (serious) adverse events and other safety related events

An **Adverse Event (AE)** is any untoward medical occurrence in a patient or a clinical investigation participant administered a pharmaceutical product and which does not necessarily have a causal relationship with the study procedure. An AE can therefore be any unfavourable and unintended sign (including an abnormal laboratory finding), symptom, or disease temporally associated with the use of a medicinal (investigational) product, whether or not related to the medicinal (investigational) product. [ICH E6 1.2]

Information on adverse events (AE) will not be collected in this Category A trial.

A **Serious Adverse Event (SAE)** is classified as any untoward medical occurrence that:

- results in death,
- is life-threatening,
- requires in-patient hospitalization or prolongation of existing hospitalisation,
- results in persistent or significant disability/incapacity, or
- is a congenital anomaly/birth defect.

In addition, important medical events that may not be immediately life-threatening or result in death, or require hospitalisation, but may jeopardise the patient or may require intervention to prevent one of the other outcomes listed above should also usually be considered serious. [ICH E2A]

SAEs will be followed until resolution or stabilisation.

Assessment of Causality

Both Investigator and Sponsor-investigator make a causality assessment of the event to the study drug, based on the criteria listed in the ICH E2A guidelines:

Relationship	Description
Definitely	Temporal relationship<br>Improvement after dechallenge*<br>Recurrence after rechallenge<br>(or other proof of drug cause)
Probably	Temporal relationship<br>Improvement after dechallenge<br>No other cause evident
Possibly	Temporal relationship<br>Other cause possible
Unlikely	Any assessable reaction that does not fulfil the above conditions
Not related	Causal relationship can be ruled out
*Improvement after dechallenge only taken into consideration, if applicable to reaction	

Unexpected Adverse Drug Reaction

An “unexpected” adverse drug reaction is an adverse reaction, the nature or severity of which is not consistent with the applicable product information (SPC).

Suspected Unexpected Serious Adverse Reactions (SUSARs)

The Sponsor-Investigator evaluates any SAE that has been reported regarding seriousness, causality and expectedness. If the event is related to the investigational product and is both serious and unexpected, it is classified as a SUSAR.

Assessment of Severity (Intensity)

Serious adverse events will be graded as either “mild”, “moderate” or “severe” according to their extent, duration (of hospitalisation), subsequent disabling/incapacitating and the required intervention(s) to prevent persistent or significant disability/incapacity.

SAEs will be documented in the clinical chart and eCRF.

10.1.2 Reporting of serious adverse events (SAE) and other safety related events

Reporting of SAEs will follow the Safety SOP for Medicinal Products of the CTU St. Gallen.

All SAEs must be reported immediately and within a maximum of 24 hours to the Sponsor-Investigator of the study. The Sponsor-Investigator will re-evaluate the SAE and return the form to the site.

SAEs resulting in death are reported to the local Ethics Committee (via local Investigator) within 7 days.

The other in the trial involved Ethics Committees receive SAEs resulting in death in Switzerland via Sponsor-Investigator within 7 days.

No SAEs will be exempted from expedited reporting. .

Reporting of SUSARs

A SUSAR needs to be reported to the local Ethics Committee (local event via local Investigator) and to Swissmedic for category B and C studies (via Sponsor-Investigator) within 7 days, if the event is fatal, or within 15 days (all other events).

The Sponsor-Investigator must inform all Investigators participating in the clinical study of the occurrence of a SUSAR. All in the trial involved Ethics Committees will be informed about SUSARs in Switzerland via Sponsor-Investigator according to the same timelines.

Reporting of Safety Signals

All suspected new risks and relevant new aspects of known adverse reactions that require safety-related measures, i.e. so called safety signals, must be reported to the Sponsor-Investigator within 24 hours. The Sponsor-Investigator must report the safety signals within 7 days to the local Ethics Committee (local event via local Investigator).

The Sponsor-Investigator must immediately inform all participating Investigators about all safety signals. The other in the trial involved Ethics Committees will be informed about safety signals in Switzerland via the Sponsor-Investigator.

Reporting and Handling of Pregnancies

Usually, a pregnancy occurring in an HIV-positive woman is a strong indication to start antiretroviral therapy as soon as possible. The drugs used in this study (NVP and 3TC) are considered safe in pregnant women and have been the standard of care in many guidelines for pregnant women. In the current protocol the exposure of the pregnant woman to HIV drugs is less than what would be the case for a standard therapy. Therefore, treatment interruption would not appear reasonable in such a case.

Exclusion criteria virtually exclude the event of a preganany during the study. However, should a pregnancy occur, during the treatment phase of the study this will be reported to the Sponsor-Investigator within 24 hours. The course and outcome of the pregnancy should be followed up carefully, and any abnormal outcome regarding the mother or the child should be documented and reported.

The sponsor investigator will discuss the proposed procedure for the individual case with the whole protocol team within 10 days with all the available treatment information depending on the situation. As an example: A woman becoming pregnant shortly before the end of the study would certainly be counselled to remain on the study regimen, since this is certainly one of the safest regimen for the first trimenon and an eventual elevation of HIV-RNA that early in the course would not increase the risk of mother to child transmission.

Periodic reporting of safety

An annual safety report will be prepared by the Sponsor-Investigator and is submitted once a year to the local Ethics Committee via local Investigators.

10.1.3 Follow up of (Serious) Adverse Events

Participants with ongoing SAEs at study termination will be further followed up until recovery or until stabilisation of the disease after termination or up to 30 days after termination. Follow-up will be done in written form or by telephone follow-up, which will be documented.If particpants with ongoing SAE will be lost to follow-up, the particpants’ general practitioner (if known) will be contacted to document the last known health status of the participant.

11. STATISTICAL METHODS

11.1 Hypothesis

Null hypothesis (H_0): Virological failure rates of bi-therapy with NVP +3TC is inferior to the best conceivable HIV maintenance strategy

Alternative hypothesis (H_I): Virological failure rates of bi-therapy with NVP +3TC is non-inferior to the best conceivable HIV-maintenance strategy which an assumed failure rate of 0%.

11.2 Determination of Sample Size

Non-inferiority of the investigational treatment regimen is given, if the upper limit of the two-sided 95 % confidence interval of the proportion with virological failure is below 4 %, i.e. nevirapine/lamivudine is less than 4 % worse than the optimum virtual control.

The estimated number of participants is n = 179 in the treatment arm, and is based on the assumption of a 0 % failure rate in an optimum (virtual) control group, the clinical assumption of a 1 % failure rate in the treatment arm, a 4 % non-inferiority margin (proposed by the FDA for HIV maintenance studies), a one-sided type I error (α) of 0.05 and a power (1-β) of 0.80. If among a total of 179 patients two will reach the primary endpoint of virologic failure, the failure rate will be 1.11% with an upper 95% confidence limit of 3.98%, which is within the accepted non-inferiority boundary. Considering the compensation of drop-outs (withdrawal of consent or discontinuation), the study requires 200 patients to be included.

The clinical assumption of a 1% failure rate is based on a pilot study with 20 patients switching to NVP/3TC bi-therapy and no evidence of treatment failure after more than 1300 observation weeks.

11.3 Statistical criteria of termination of trial

Stopping rule for individual participants:

Any patient with virological failure (HIV-RNA ≥200 cp/ml, confirmed by a second measurement within 4 weeks) will be promptly informed, and therapy with the investigational treatment regime will be discontinued. The patients concerned will be switched to a treatment as deemed adequate by the principle investigator at the study sites (see section 7.3).

Stopping rule for the study:

If three or more patients demonstrate true virological failure (with development of resistance) at any time during the study, the study will be terminated prematurely in order to prevent the occurrence of more virological failures with possible consecutive development of resistance.

11.4 Planned Analyses

11.4.1 Datasets to be analysed, analysis populations

For the primary and the secondary outcomes all eligible subjects will be included in the analysis. Non virological treatment failures will be compared not only with a virtual control group representing the optimum outcome (0 % non virological failure rate) but also with a subset of SHCS patients currently and for more than 6 months on a NVP based treatment regimen with viral loads <200 HIV-RNA cp/ml.

For the analysis of possible markers for active viral replication, a subset of participants, i.e. who will be recruited and whose blood samples will be subjected to laboratory analysis at the centre in St. Gallen, will be included. Based on the similar analysis from the MOST study (3.1.6), 60 patients would be sufficient to detect a significant increase in activation markers.

11.4.2 Primary Analysis

The primary analysis will compare the proportion of patients with HIV-RNA ≥ 200 cp/ml at week 48 (confirmed by a second measurement within 4 weeks, snapshot approach) with the virtual control group in which the proportion of participants with virological failure is 0 % (i.e. the optimum outcome in any cART). The 95 %-confidence interval of the proportion in the treatment group is calculated and the upper limit compared to the pre-specified non-inferiority margin (4 %, which is equal to the difference between the failure rate in the treatment arm and 0% failure rate in the control group).

The primary analysis will be performed by the protocol team under the lead of the trial statistician.

11.4.3 Definitions of virological outcome

Virological outcome at week 48 will be categorized as suggested by the FDA snapshot approach:

- HIV-RNA <200 cp/ml
- HIV-RNA ≥200 cp/ml (confirmed by second test within 4 weeks, includes patients who discontinued study drug/study before week 48 for lack of efficacy)
- No virological data at week 48:

- Discontinued study/study drug due to AE or Death
- Discontinued study/study drug for Other Reasons
- On study but missing data in window

The snapshot approach only uses HIV-RNA data at the visit (window period) of interest. For the week 48-visit, the window (through end-of-study week) is week 42-54, corresponding to day 295-378.

11.4.4 Principles of snapshot analysis

This method follows a *Virology First* hierarchy.

This means that the hierarchy for assessing the outcome is HIV-RNA <200 cp/ml, or HIV-RNA ≥200 cp/ml, first, for any given time window followed by reasons for *No Virological Data in the 48-Week Window*.

Percentages not included in the HIV-RNA below or equal to or above 200 cp/ml rows should describe reasons for no data at a specified analysis time window.

11.4.5 Procedures for calculating virological outcome

Virological data in the 48-Week Window (Day 295-378)

Virological outcome should be determined by the last available measurement while the patient is on treatment and continued on trial within the time window.

Transient blips of HIV-RNA >50 cp/ml occur for a variety of reasons and this does not always signify true virological failure to the regimen. True virological failure may only be determined after assessment of drug adherence, repeat HIV-RNA testing with continued treatment, and/or resistance testing. Snapshot time windows allow time for clinical assessment and retesting to reduce the number of patients counted as having HIV-RNA levels above 50 cp/ml, because of transient blips.

Examples:

1) HIV-RNA = 580 copies/ml at Day 336, HIV-RNA <50 cp/ml at Day 350. This is categorized as HIV-RNA <200 cp/ml.
2) In the rare example that someone would have HIV-RNA <50 cp/ml at Day 336 and then ≥200 cp/ml at Day 350, the result would be counted as ≥200 cp/ml (if confirmed).

No Virological data in the 48-Week Window (Day 295-378)

There are three main reasons for no data in the window:

I. Discontinued study/study drug due to Adverse Event or Death.

Any patient who discontinues because of AE or death **before** the window is classified as *Discontinued due to AE or Death* (as appropriate), regardless of the HIV-RNA result, even if the HIV-RNA is <200 cp/ml at the time of discontinuation. However, if a patient has an HIV-RNA value **in the time window** and also discontinues after the viral load was tested in the time window, the viral load data are used to classify the patient's response. This is the Virology First hierarchy.

Examples:

1) HIV-RNA <200 cp/ml at Day 336 and discontinues due to AE or even dies on Day 360 – this person is categorized as having HIV-RNA <200 cp/ml.
2) If HIV-RNA is 552 cp/ml on Day 336 and the patient discontinues on Day 360, the patient is categorized as having HIV-RNA ≥200 cp/ml.

II. Discontinued study/study drug for Other Reasons.

The examples above also apply to this category. If a patient discontinues the study before the window because of *lack of efficacy* then the patient is included in the HIV-RNA ≥200 cp/ml-category and not in the *Discontinued for Other Reasons*-category. To further clarify, for patients who discontinued for Other Reasons, it is important to realize that in the Virology First hierarchy only patients who have achieved virological suppression can be counted as Discontinued for Other Reasons.

Examples:

1) If a patient discontinues because the *subject withdrew consent* and his or her HIV-RNA at the time of discontinuation was ≥200 cp/ml, then he or she is categorized as HIV-RNA ≥200 cp/ml and NOT as Discontinued for Other Reasons.
2) However, if a patient discontinued because of *Lost to Follow-Up* and the last HIV-RNA result was 49 cp/ml, then the patient can be categorized as Discontinued for Other Reasons.

III. On study but missing data in window.

Only data in the window can be used for patients remaining on study.

Examples:

1) If there are no data during Days 295 to 378, but there is an HIV-RNA <200 cp/ml on Day 380, this patient is considered *On Study but Missing Data in Window*. This patient can count as <200 cp/ml at

subsequent analysis points (e.g., 96 weeks), if he or she remains undetectable at the subsequent analysis window (e.g., 96 weeks).

2) Conversely, if there are no data during Days 295 to 378, but there is an HIV-RNA ≥200 on Day 280, this patient is also classified as On Study but Missing Data in Window.

Thus, patients stopping NVP/3TC bi-therapy due to other reasons than virological failure, e.g. death, adverse reactions, loss to follow-up, withdrawal of consent (patient wish) are not considered as virological failures as long as they were virologically suppressed (<200 cp/ml) at their last visit (described in FDA-snapshot approach, p.21 ref. 36)

11.4.6 Secondary Analyses

The analysis of the secondary endpoints is performed at the end of the study by the project team, lead by the study biostatistician, Andrea Bregenzer.

The following signs of potentially reduced antiretroviral activity will be analysed descriptively:

- Increase in frequency of low level HIV-RNA detection
- Increase in HIV reservoir size
- Increase in immune activation markers in CD8+ T-cells

-

Analysis of low level HIV-RNA replication/viral blips

Low level detectable HIV-RNA might be interpreted as decreased antiviral activity. For the purpose of this evaluation, the following categories of low-level HIV-RNA detection will be used: 20-49; 50-99; 100-199 cp/ml.

Analysis of HIV reservoir and immune activation

Assessment of HIV-reservoir size and immune activation marker is described in detail in section 9.2.2.

11.4.7 Interim analyses

No interim analyses planned. The virological (and non virological) failure rate is (are) documented in an ongoing manner in order to immediately detect possible treatment failures.

11.4.8 Safety analysis

Not applicable.

11.4.9 Deviation(s) from the original statistical plan

Any deviation from the planned analyses regarding the primary and secondary outcomes will be based on discussions within the protocol team and reported in the final analysis.

11.5 Handling of missing data and drop-outs

Handling of drop-outs is described in 8.5.

Handling of missing data

If a patient does not appear for the confirmatory visit in case of an HIV-RNA value ≥ 50 cp/ml, the initially obtained viral load measurement will be considered as confirmed.

12. QUALITY ASSURANCE AND CONTROL

12.1 Data handling and record keeping / archiving

Data are handled and related documents are archived according to existing KSSG SOPs.

12.1.1 Case Report Forms

The study will use electronic Case Report Forms set up by the sponsor team (e-CRF) and provided by the CTU of the KSSG using the SecuTrial® system) for recording all study-related data of the patient.

Study-related data of the patient will be collected in a coded manner. The names of the patients will not be disclosed on the e-CRF. A code (unique) will be attributed to each patient registered.

All study data will be entered in the Electronic Data Capture (EDC, SecuTrial®) system by the study nurse or the local data manager with no double data entry.

Persons authorized by the sponsor-investigator to perform data entry or data review will be communicated to the data manager who will provide individual access codes according to the function assigned. CRF data entry authorization will be documented on each delegation log and on a list of all authorized persons and their function stored with the data manager.

12.1.2 Specification of source documents

The source database will be the clinical chart at the study sites and includes demographic data, participation in the study, visit dates, Informed Consent Forms, HIV treatment history,clinical data (medical history), SAEs and concomitant therapies of the participants. Laboratory data related to the study are stored with the clinical chart at the study site.

The study administration will provide printed CRF worksheets for the screening visit that can either be used as checklists to ensure complete data capturing or be used as primary source files depending on the data organization on site. In the latter case, the CRF (source document) must be stored with the local primary data.

12.1.3 Record keeping / archiving

All study data must be archived for a minimum of 10 years after study termination or premature termination of the clinical trial. The study files will be stored in the study archive of the KSSG, Division of Infectious Diseases.

12.2 Data management

12.2.1 Data Management System

The CRFs in this trial are implemented electronically using a dedicated electronic data capturing (EDC) system (SecuTrial®). The EDC system is activated for the trial only after successfully passing a formal test procedure. All data entered in the e-CRFs are stored on a Windows server in a dedicated Oracle exadata database. Responsibility for hosting the EDC system and the database lies with the CTU of the KSSG.

12.2.2 Data security, access and back-up

The server hosting the EDC system and the database is kept in a locked server-room. Only the system administrators have direct access to the server. A role concept with personal passwords (site investigator, statistician, monitor, administrator etc.) regulates permission for each user to use the system and database as he/she requires.

All data entered into the e-CRFs are transferred to the database using Secure Sockets Layer (SSL) encryption. Each data point has attributes attached to it identifying the user who entered it with the exact time and date. Retrospective alterations of data in the database are recorded in an audit table. Time, table, data field and altered value, and the person are recorded (audit trail). A multi-level back-up system is implemented.

12.2.3 Analysis and archiving

Data extraction for analysis will be performed by the CTU and extracted data will be made available to the sponsor investigator in Excel format. The data analysis files will be stored on a CD (Backup, to be filed in the TMF) for a minimum of 10 years.

12.2.4 Electronic and central data validation

Data are checked by the EDC system for completeness and plausibility after termination of data entry for each participant. In addition, central data reviews will be performed on a regular basis to ensure completeness of the data collected and accuracy of the primary outcome data. PIs will receive a request for data updates in case of missing or questionable variables. Local monitoring will compare the electronic data entry with local data in primary source files.

Before the database is locked, the PI will validate the collected data with his signature.

12.3 Monitoring

For quality control of the study conduct and data retrieval, all study sites will be visited by appropriately trained and qualified monitors (CTU at KSSG) according to the monitoring plan. Monitoring will be conducted by the CTU according to CTU SOPs. Any findings and comments will be documented in site visit reports and communicated to the local Investigator and to the Sponsor as applicable. The investigator and his delegates at the participating study sites will support the monitor in his / her activities. Prior to study start (first participant enrolled), a plan detailing all monitoring-related procedures will be developed (monitoring plan). All source data and relevant documents will be accessible to Monitors and questions of Monitors are answered during site visits.

12.4 Audits and Inspections

The study documentation and the source data/documents are accessible to auditors/inspectors (CEC) and questions are answered during inspections. All involved parties must keep the participant data strictly confidential. The sponsor-investigator does not plan an audit or inspection of the trial conduct.

12.5 Confidentiality, Data Protection

Direct access to source documents will be permitted for the purposes of monitoring, audits and inspections. Both ethics committee members and employees must also understand the confidentiality requirements for any information divulged to them. The data generated by this study will be considered confidential by the investigators, except to the extent that it is included in a publication as agreed in the publication policy of this protocol.

All data will be collected without any names and identifying information will not be recorded in the study data files. List of names with study identifiers will be kept at the individual clinical sites.

The protocol will be accessible to the trial team and all investigators and delegates during and after the course of the study. The dataset will be accessible only to the data manager, the principal investigators, sponsor-investigator and the statistician.

12.6 Storage of biological material and related health data

No storage of biological material related health data is planned. Plasma and PBMCs are only taken as outlined in the protocol to determine HIV-RNA (plasma) or HIV-DNA (PBMCs).

13. PUBLICATION AND DISSEMINATION POLICY

No patient data will be presented in scientific meetings or publications that could permit identification of any individual study participant. Publication of data derived from this protocol will be supervised by the Sponsor-Investigator. No other publication will be made before the primary manuscript has been agreed upon by all co-investigators. The main funding source has no role in data management, in the analysis plan, in the decision what and where to submit for publication and in the formulation of the conclusions.

14. FUNDING AND SUPPORT

14.1 Funding

The study has been submitted to the Swiss National Science Foundation (SNF) for funding (revised version) for funding. The SNF decision is due end of March 2019. A grant has been approved by the research commission ("Forschungskommission") of the cantonal hospital of St. Gallen for funding work preceding the study initiation in April 2019.

14.2 Other Support

Not applicable.

15. INSURANCE

For category A studies no special insurance is required.

16. REFERENCES

1. Bierman WFW, van Agtmael MA, Nijhuis M, Danner SA, Boucher CAB. HIV monotherapy with ritonavir-boosted protease inhibitors: a systematic review. AIDS Lond Engl. 28. Januar 2009;23(3):279–91.

2. Brenner BG, Wainberg MA. Clinical benefit of dolutegravir in HIV-1 management related to the high genetic barrier to drug resistance. Virus Res. 15. Juli 2017;239:1–9.

3. D'Abbraccio M, Busto A, De Marco M, Figoni M, Maddaloni A, Abrescia N. Efficacy and Tolerability of Integrase Inhibitors in Antiretroviral-Naive Patients. AIDS Rev. September 2015;17(3):171–85.

4. Kahlert C, Bregenzer A, Gutmann C, Otterbech S, Hoffmann M, Schmid P, u. a. Late treatment failures in cerebrospinal fluid in patients on long-term maintenance ART with ritonavir-boosted protease PI monotherapy. Infection. Juni 2016;44(3):329–35.

5. Crespo M, Navarro J, Martinez-Rebollar M, Podzamczer D, Domingo P, Mallolas J, u. a. Improvement of BMD after Switching from Lopinavir/R Plus Two Nucleos(T)ide Reverse Transcriptase Inhibitors to Lopinavir/R Plus Lamivudine: OLE-LIP Substudy. HIV Clin Trials. Mai 2016;17(3):89–95.

6. Arribas JR, Girard P-M, Landman R, Pich J, Mallolas J, Martínez-Rebollar M, u. a. Dual treatment with lopinavir-ritonavir plus lamivudine versus triple treatment with lopinavir-ritonavir plus lamivudine or emtricitabine and a second nucleos(t)ide reverse transcriptase inhibitor for maintenance of HIV-1 viral suppression (OLE): a randomised, open-label, non-inferiority trial. Lancet Infect Dis. Juli 2015;15(7):785–92.

7. Mathis S, Khanlari B, Pulido F, Schechter M, Negredo E, Nelson M, u. a. Effectiveness of Protease Inhibitor Monotherapy versus Combination Antiretroviral Maintenance Therapy: A Meta-Analysis. PLoS ONE. 19. Juli 2011;6(7):e22003.

8. Finzi D, Blankson J, Siliciano JD, Margolick JB, Chadwick K, Pierson T, u. a. Latent infection of CD4+ T cells provides a mechanism for lifelong persistence of HIV-1, even in patients on effective combination therapy. Nat Med. Mai 1999;5(5):512–7.

9. Riddell J. 2018 IAS-USA Recommendations for the Use of Antiretroviral Therapy for HIV: Building on Decades of Progress. JAMA. 24. Juli 2018;320(4):347.

10. d'Ettorre G, Baroncelli S, Micci L, Ceccarelli G, Andreotti M, Sharma P, u. a. Reconstitution of intestinal CD4 and Th17 T cells in antiretroviral therapy suppressed HIV-infected subjects: implication for residual immune activation from the results of a clinical trial. PloS One. 2014;9(10):e109791.

11. Joos B, Fischer M, Kuster H, Pillai SK, Wong JK, Böni J, u. a. HIV rebounds from latently infected cells, rather than from continuing low-level replication. Proc Natl Acad Sci. 28. Oktober 2008;105(43):16725–30.

12. Gueler A, Moser A, Calmy A, Günthard HF, Bernasconi E, Furrer H, u. a. Life expectancy in HIV-positive persons in Switzerland: matched comparison with general population. AIDS Lond Engl. 7. November 2016;

13. Ryom L, Boesecke C, Gisler V, Manzardo C, Rockstroh JK, Puoti M, u. a. Essentials from the 2015 European AIDS Clinical Society (EACS) guidelines for the treatment of adult HIV-positive persons. HIV Med. Februar 2016;17(2):83–8.

14. Perelson AS, Neumann AU, Markowitz M, Leonard JM, Ho DD. HIV-1 dynamics in vivo: virion clearance rate, infected cell life-span, and viral generation time. Science. 15. März 1996;271(5255):1582–6.

15. Mansky LM, Temin HM. Lower in vivo mutation rate of human immunodeficiency virus type 1 than that predicted from the fidelity of purified reverse transcriptase. J Virol. August 1995;69(8):5087–94.

16. Coffin JM. HIV population dynamics in vivo: implications for genetic variation, pathogenesis, and therapy. Science. 27. Januar 1995;267(5197):483–9.

17. Hukezalie KR, Thumati NR, Côté HCF, Wong JMY. In vitro and ex vivo inhibition of human telomerase by anti-HIV nucleoside reverse transcriptase inhibitors (NRTIs) but not by non-NRTIs. PloS One. 2012;7(11):e47505.

18. Leeansyah E, Cameron PU, Solomon A, Tennakoon S, Velayudham P, Gouillou M, u. a. Inhibition of telomerase activity by human immunodeficiency virus (HIV) nucleos(t)ide reverse transcriptase inhibitors: a potential factor contributing to HIV-associated accelerated aging. J Infect Dis. April 2013;207(7):1157–65.

19. Bierman WFW, van Agtmael MA, Nijhuis M, Danner SA, Boucher CAB. HIV monotherapy with ritonavir-boosted protease inhibitors: a systematic review. AIDS Lond Engl. 28. Januar 2009;23(3):279–91.

20. Shuter J. Forgiveness of non-adherence to HIV-1 antiretroviral therapy. J Antimicrob Chemother. April 2008;61(4):769–73.

21. Baril J-G, Angel JB, Gill MJ, Gathe J, Cahn P, van Wyk J, u. a. Dual Therapy Treatment Strategies for the Management of Patients Infected with HIV: A Systematic Review of Current Evidence in ARV-Naive or ARV-Experienced, Virologically Suppressed Patients. PloS One. 2016;11(2):e0148231.

22. Bergersen BM. Cardiovascular risk in patients with HIV Infection: impact of antiretroviral therapy. Drugs. 2006;66(15):1971–87.

23. Bundesamt für Gesundheit. NPHS: National Programm on HIV and STDs. accessible on www.bag.admin.ch [Internet]. [zitiert 1. Oktober 2017]. Verfügbar unter: https://www.bag.admin.ch/bag/de/home/themen/mensch-gesundheit/uebertragbare-krankheiten/infektionskrankheiten-a-z/hiv.html

24. Rodríguez-Arrondo F, Aguirrebengoa K, Portu J, Muñoz J, García MA, Goikoetxea J, u. a. Long-term effectiveness and safety outcomes in HIV-1-infected patients after a median time of 6 years on nevirapine. Curr HIV Res. September 2009;7(5):526–32.

25. Kamara DA, Smith C, Ryom L, Reiss P, Rickenbach M, Phillips A, u. a. Longitudinal analysis of the associations between antiretroviral therapy, viraemia and immunosuppression with lipid levels: the D:A:D study. Antivir Ther. 2016;21(6):495–506.

26. Wit FWNM, Kesselring AM, Gras L, Richter C, van der Ende ME, Brinkman K, u. a. Discontinuation of nevirapine because of hypersensitivity reactions in patients with prior treatment experience, compared with treatment-naive patients: the ATHENA cohort study. Clin Infect Dis Off Publ Infect Dis Soc Am. 15. März 2008;46(6):933–40.

27. Patterson P, Socías E, Pryluka D, Lapadula P, Pérez H, Cahn P. Switching to nevirapine-based regimens after undetectable viral load is not associated with increased risk of discontinuation due to toxicity. J Int AIDS Soc. 2014;17(4 Suppl 3):19794.

28. Calcagno A, Di Perri G, Bonora S. Pharmacokinetics and pharmacodynamics of antiretrovirals in the central nervous system. Clin Pharmacokinet. Oktober 2014;53(10):891–906.

29. Non-Inferiority Clinical Trials To Establish Effectiveness; Guidance for Industry; Availability [Internet]. Federal Register. 2016 [zitiert 1. Oktober 2017]. Verfügbar unter: https://www.federalregister.gov/documents/2016/11/08/2016-26931/non-inferiority-clinical-trials-to-establish-effectiveness-guidance-for-industry-availability

30. Trelle S. Exploratory trials in mental health: anything to learn from other disciplines? Evid Based Ment Health. Februar 2017;20(1):21–4.

31. Kahlert C, Hupfer M, Wagels T, Bueche D, Fierz W, Walker UA, u. a. Ritonavir boosted indinavir treatment as a simplified maintenance „mono"-therapy for HIV infection. AIDS Lond Engl. 9. April 2004;18(6):955–7.

32. Eron JJ, Vernazza PL, Johnston DM, Seillier-Moiseiwitsch F, Alcorn TM, Fiscus SA, u. a. Resistance of HIV-1 to antiretroviral agents in blood and seminal plasma: implications for transmission. AIDS Lond Engl. 22. Oktober 1998;12(15):F181-9.

33. Vernazza P, Daneel S, Schiffer V, Decosterd L, Fierz W, Klimkait T, u. a. The role of compartment penetration in PI-monotherapy: the Atazanavir-Ritonavir Monomaintenance (ATARITMO) Trial. AIDS Lond Engl. 19. Juni 2007;21(10):1309–15.

34. Gutmann C, Cusini A, Günthard HF, Fux C, Hirschel B, Decosterd L-A, u. a. Randomized controlled study demonstrating failure of LPV/r monotherapy in HIV: the role of compartment and CD4-nadir. AIDS Lond Engl. 24. September 2010;24(15):2347–54.

35. Kahlert, CR, Cipriani, M., Schmid, P., Vernazza, PL. Nevirapine plus lamivudine maintain HIV-1 suppression trhough week 48. In Amsterdam; 2018 [zitiert 15. September 2018]. Verfügbar unter: http://tiny.cc/NVP

36. Human Immunodeficiency Virus-1 Infection: Developing Antiretroviral Drugs for Treatment Guidance for

Industry. 2015;47.

17. APPENDICES

1. SPCs Nevirapine (Nevirapine-Mepha Retardtabletten®) and Lamivudine (3TC®)
