# Supplementary material for: A novel design using a virtual control group to evaluate non-inferiority of nevirapine and lamivudine dual maintenance in HIV therapy
Source: PLoS One. 2026 Jul 8;21(7):e0351576. doi: 10.1371/journal.pone.0351576 (PMC13345260; doi:10.1371/journal.pone.0351576)
Supplement: Attachment — Submitted filename: Response to Reviewers PLOS ONE.docx [file pone.0351576.s007.docx]

**Response to Reviewers PLOS ONE 05/2026**

Dear Professor De Socio and Reviewers,

We thank the involved professionals for their careful reading and concise proposals for improving the manuscript. We have revised the manuscript accordingly and believe these changes have substantially improved the quality and clarity of the work. Below, we respond to each comment in detail. Comments from the reviewers and the editor are in regular font, while the authors’ responses are in italic and bold.

**Overview of the most important revisions**

- Adaptation of formation and file naming to meet PLOS ONE style requirements
- Revision of the statistical analyses as suggested by both reviewers
- Modifications in all sections of the manuscript as suggested by the reviewers

**Editor comments**

When submitting your revision, we need you to address these additional requirements.

1. Please ensure that your manuscript meets PLOS ONE's style requirements, including those for file naming. The PLOS ONE style templates can be found at

<https://journals.plos.org/plosone/s/file?id=wjVg/PLOSOne_formatting_sample_main_body.pdf> and

[https://journals.plos.org/plosone/s/file?id=ba62/PLOSOne_formatting_sample_title_authors_affiliations.pdf](https://track.editorialmanager.com/CL0/https:%2F%2Fjournals.plos.org%2Fplosone%2Fs%2Ffile%3Fid=ba62%2FPLOSOne_formatting_sample_title_authors_affiliations.pdf/1/010f019be3e0fafd-534e73a0-8dd2-4aec-b141-5540b5f64688-000000/T50Sr5eOumXyLQJO0tKCv-nD-3a5aNz3h209LuuT5hM=247)

***We implemented several adjustments, such as renaming figure files, to ensure compliance with the style guidelines. As requested in the guidelines, we have formatted the manuscript with double spacing.***

2. Please include a complete copy of PLOS’ questionnaire on inclusivity in global research in your revised manuscript. Our policy for research in this area aims to improve transparency in the reporting of research performed outside of researchers’ own country or community. The policy applies to researchers who have travelled to a different country to conduct research, research with Indigenous populations or their lands, and research on cultural artefacts. The questionnaire can also be requested at the journal’s discretion for any other submissions, even if these conditions are not met. Please find more information on the policy and a link to download a blank copy of the questionnaire here: <https://journals.plos.org/plosone/s/best-practices-in-research-reporting>. Please upload a completed version of your questionnaire as Supporting Information when you resubmit your manuscript.

**We uploaded the questionnaire as Supporting Information.**

3. We note that the grant information you provided in the ‘Funding Information’ and ‘Financial Disclosure’ sections do not match.

When you resubmit, please ensure that you provide the correct grant numbers for the awards you received for your study in the ‘Funding Information’ section.

**We made an adaptation at the end of the manuscript. The Financial Disclosure section appears to be locked, as the corresponding author cannot edit this section during the revision process on the PLOS ONE website.**

4. When completing the data availability statement of the submission form, you indicated that you will make your data available on acceptance. We strongly recommend all authors decide on a data sharing plan before acceptance, as the process can be lengthy and hold up publication timelines. Please note that, though access restrictions are acceptable now, your entire data will need to be made freely accessible if your manuscript is accepted for publication. This policy applies to all data except where public deposition would breach compliance with the protocol approved by your research ethics board. If you are unable to adhere to our open data policy, please kindly revise your statement to explain your reasoning and we will seek the editor's input on an exemption. Please be assured that, once you have provided your new statement, the assessment of your exemption will not hold up the peer review process.

**We are currently preparing the data file and will make the data available on Zenodo, if the manuscript is accepted for publication.**

5. If the reviewer comments include a recommendation to cite specific previously published works, please review and evaluate these publications to determine whether they are relevant and should be cited. There is no requirement to cite these works unless the editor has indicated otherwise.

**Reviewer 1**

This is a somewhat dated analysis of the virological response to dual therapy (2DR) with nevirapine (NVP) and lamivudine (3TC) in people who previously achieved viral suppression on 3DR containing nevirapine and the results have limited clinical applicability. The rationale is that once people who can tolerate NVP are identified then NVP-based 2DR is a viable long-term option for these individuals. Although most people now use INSTI-based dual therapy in this setting NVP-based 2DR are still an alternative option for a minority of people who do not wish to change to INSTI. I have several points that need to be addressed

Main points

1.This is essentially a mono-arm observational study which is sold as a clinical trial. I do not really see the need or advantage to call it a trial with a comparator “virtual control arm” that has 100% efficacy. Indeed, the procedure might be conservative, but strictly speaking because the control arm is only virtual with no simulated data being created, recommended tests (e.g. the Farrington -Manning score) cannot be performed in this study and the simple calculation of a proportion with a Clopper-Pearson 95% CI cannot be used to establish non-inferiority to the virtual arm. It would have been more informative to show it as a cohort with maximum 96 weeks follow-up and show the incidence of virological as well as treatment failure at different time-points. To this extent it is unclear why it was not possible to follow-up this cohort to more recent times. A 3-year estimate of the proportion still receiving the 2DR NVP-based regimen with HIV-RNA<50 copies/mL would have been a valuable information.

***We appreciate the detailed comment of the reviewer. We have now simulated data for an ideal control group consisting of 194 patients with no observed virological failures, and we performed the Farrington-Manning-Test for non-inferiority of DT as suggested. We also applied the same approach for the post-study observation. We have decided not to simulate a virtual control group using the `rbinom` function from the R base package, since non-inferiority is demonstrated in all cases where X0 > 0. In addition, an arbitrary yet justified assumption about the exact failure rate in the control group would need to be made in order to obtain a distribution.***

***While we agree that a 3-year estimate of the proportion of participants with HIV-RNA <50 cp/ml would further enhance the study, the multicenter design makes extended data collection challenging. Consequently, the study period was defined as 48 weeks following FDA guidelines for HIV maintenance therapy, supplemented by a post-study observation of up to 12 months. However, we additionally re-contacted the other centers to supplement further results, ensuring that there were no missing data during the 12-month post-study observation period. Regarding an extended follow-up, we are planning follow-up studies to assess long-term safety and the impact of this dual therapy on the viral reservoir, as noted at the end of the discussion.***

2.Indeed, being a maintenance study, the main outcome should be durability and not virological failure. Suggest that the authors use a time to treatment failure (TF) approach counting as events: i) virological failures, ii) all discontinuations regarding of the reason and iii) death.

***Focusing on durability is an interesting consideration. However, our study was originally designed in accordance with FDA guidelines, with virological failure as the primary endpoint. Since our study was designed in alignment with other HIV non-inferiority trials, the proposed approach (TF) is not feasible. In the assessment of HIV maintenance therapy, the primary focus is on virological suppression; other events or discontinuations are counted only if patients are not virologically suppressed. All discontinuations and deaths were carefully reviewed for any potential association with the dual therapy, and no such correlation was observed. We have updated the Methods section to specify that treatment failure refers to virological failure.***

3. Seven premature discontinuations have been excluded. There should be at least one TF analysis in which these are instead counted as events. At least the 4 discontinuations due to SAE and the single drug to drug interactions should be events. There were also 2 deaths which occurred during the study period (I assume these were among the 194 included in the analysis).

***In line with the study design based on the guidelines mentioned above, participants were required to complete the 48-week period to be included in the final analysis (i.e., 194 patients; Fig. 1). The deaths would only have been included, if virological failure occurred during the study period among these patients. The circumstances of the death events are described in the Results section.***

4. Using TF as primary endpoint with 7 failures and 201 participants the incidence rate is 3.5% with exact limits of (1.4-7.0%). Thus, even keeping the novel approach for a non-inferiority trial design, data clearly fail to show non-inferiority when using a more relevant clinical endpoint.

***The FDA-defined non-inferiority margin of 4% was used to demonstrate non-inferiority specifically for the primary endpoint of virological failure. Using the TF approach would make it challenging to determine a valid non-inferiority margin, particularly when comparing the results to a perfect virtual control group with no events. If the TF approach was applied, a different study design with a real control group, for example a randomized controlled trial, would be more appropriate.***

Minor points:

1.Bottom of page 2. Occasional viral blips are described as HIV-RNA measurements of 50-200 cp/ml followed by an additional measurement of <50 cp/ml. This is not an accurate definition of blip. It should be a re-suppression to a value of <50 cp/ml but without any change in ART (certainly without a change of NVP).

***Thank you for your input; we have revised the sentence as suggested.***

2. Page 4. I would avoid expressions such as “median change was not significant” presumably linked to the use of a 5% threshold for the p-value. Authors should report in terms of strength of evidence against the null.

***We appreciate this comment. In the manuscript, we reported the exact p-value as a measure of evidence against the null hypothesis. For further clarification, we have now explicitly specified the null hypothesis.***

3. Page 5 Discussion. There is no reference to the last part of this sentence “Despite NVP having a low resistance barrier and requiring only a single mutation in the reverse transcriptase genome to develop resistance(15), DT with 3TC proved to be an efficient combination to achieve constant viral suppression”. In general, it seems out of place as the study evaluates maintenance of suppression not initial achievement.

***We have rephrased the final part of the sentence. Since the statement is based on our study, no external reference is necessary in our opinion.***

**Reviewer 2**

This study presents an interesting multicenter non-inferiority trial investigating the efficacy of a switch strategy combining nevirapine and lamivudine in patients on cART with sustained HIV plasma viral load (pVL) <50 copies/mL for at least two years. The Methods section is clearly described; however, the statistical analysis would benefit from a thorough review by a biostatistics expert. The Results section is well written, although I have a few minor concerns:

- In the Introduction, the authors state that “NVP is the only NNRTI that does not negatively impact lipid levels.” However, the effect on lipid profiles was not evaluated in this study. Including such an analysis could strengthen the rationale for choosing a regimen with a lower resistance barrier compared to currently available dual therapies, particularly those based on integrase inhibitors (INIs).

***Thank you for your valuable review and suggestions. Considering your suggestions, those of Reviewer 1 and in addition to the supplementary analysis, the manuscript has undergone a thorough review by a statistician (see Acknowledgements).***

***Furthermore, we agree with the reviewer that analyzing changes in the lipid profile could provide an additional reason for choosing this dual therapy. However, lipid parameters were available only from certain centers (i.e. below 50% of the whole population); therefore, any conclusions drawn from these data would have limited informative value. However, information on this point is available from the literature which is cited, and it is mentioned in the manuscript as a limitation. We now explicitly refer to lipid levels as an example.***

-Darunavir/ritonavir/dolutegravir should not be considered a triple therapy.

***Thank you for the clarification. We have revised the sentence accordingly.***

The Discussion is well articulated, with an appropriate number of references. Moreover, the study limitations are clearly acknowledged.
