# Supplementary material for: A novel design using a virtual control group to evaluate non-inferiority of nevirapine and lamivudine dual maintenance in HIV therapy
Source: PLoS One. 2026 Jul 8;21(7):e0351576. doi: 10.1371/journal.pone.0351576 (PMC13345260; doi:10.1371/journal.pone.0351576)
Supplement: Peer review history

# Author comment: Author response to Decision Letter 0

**DOI:** 10.1371/journal.pone.0351576.r001

**Transfer Alert**

This paper was transferred from another journal. As a result, its full editorial history (including decision letters, peer reviews and author responses) may not be present.

28 Nov 2025

# Peer review: Decision Letter 0

**DOI:** 10.1371/journal.pone.0351576.r002

**Authors:** Giuseppe De Socio

21 Jan 2026

PONE-D-25-54404

A Novel Design using a Virtual Control to evaluate Non-inferiority of Nevirapine and Lamivudine Dual Maintenance in HIV therapy

PLOS One

Dear Dr. Suter,

Thank you for submitting your manuscript to PLOS ONE. After careful consideration, we feel that it has merit but does not fully meet PLOS ONE’s publication criteria as it currently stands. Therefore, we invite you to submit a revised version of the manuscript that addresses the points raised during the review process.

Please submit your revised manuscript by the next Mar 07 2026 11:59PM. If you will need more time than this to complete your revisions, please reply to this message or contact the journal office at plosone@plos.org. When you're ready to submit your revision, log on to https://www.editorialmanager.com/pone/ and select the 'Submissions Needing Revision' folder to locate your manuscript file.

Please include the following items when submitting your revised manuscript:

- A letter that responds to each point raised by the academic editor and reviewer(s). You should upload this letter as a separate file labeled 'Response to Reviewers'.
- A marked-up copy of your manuscript that highlights changes made to the original version. You should upload this as a separate file labeled 'Revised Manuscript with Track Changes'.
- An unmarked version of your revised paper without tracked changes. You should upload this as a separate file labeled 'Manuscript'.

If you would like to make changes to your financial disclosure, please include your updated statement in your cover letter. Guidelines for resubmitting your figure files are available below the reviewer comments at the end of this letter.

If applicable, we recommend that you deposit your laboratory protocols in protocols.io to enhance the reproducibility of your results. Protocols.io assigns your protocol its own identifier (DOI) so that it can be cited independently in the future. For instructions see: https://journals.plos.org/plosone/s/submission-guidelines#loc-laboratory-protocols. Additionally, PLOS ONE offers an option for publishing peer-reviewed Lab Protocol articles, which describe protocols hosted on protocols.io. Read more information on sharing protocols at https://plos.org/protocols?utm_medium=editorial-email&utm_source=authorletters&utm_campaign=protocols.

We look forward to receiving your revised manuscript.

Kind regards,

Giuseppe Vittorio De Socio, MD, PhD

Academic Editor

PLOS One

Journal Requirements:

When submitting your revision, we need you to address these additional requirements.

1. Please ensure that your manuscript meets PLOS ONE's style requirements, including those for file naming. The PLOS ONE style templates can be found at

https://journals.plos.org/plosone/s/file?id=wjVg/PLOSOne_formatting_sample_main_body.pdf and

https://journals.plos.org/plosone/s/file?id=ba62/PLOSOne_formatting_sample_title_authors_affiliations.pdf

2. Please include a complete copy of PLOS’ questionnaire on inclusivity in global research in your revised manuscript. Our policy for research in this area aims to improve transparency in the reporting of research performed outside of researchers’ own country or community. The policy applies to researchers who have travelled to a different country to conduct research, research with Indigenous populations or their lands, and research on cultural artefacts. The questionnaire can also be requested at the journal’s discretion for any other submissions, even if these conditions are not met. Please find more information on the policy and a link to download a blank copy of the questionnaire here: https://journals.plos.org/plosone/s/best-practices-in-research-reporting. Please upload a completed version of your questionnaire as Supporting Information when you resubmit your manuscript.

3. We note that the grant information you provided in the ‘Funding Information’ and ‘Financial Disclosure’ sections do not match.

When you resubmit, please ensure that you provide the correct grant numbers for the awards you received for your study in the ‘Funding Information’ section.

4. When completing the data availability statement of the submission form, you indicated that you will make your data available on acceptance. We strongly recommend all authors decide on a data sharing plan before acceptance, as the process can be lengthy and hold up publication timelines. Please note that, though access restrictions are acceptable now, your entire data will need to be made freely accessible if your manuscript is accepted for publication. This policy applies to all data except where public deposition would breach compliance with the protocol approved by your research ethics board. If you are unable to adhere to our open data policy, please kindly revise your statement to explain your reasoning and we will seek the editor's input on an exemption. Please be assured that, once you have provided your new statement, the assessment of your exemption will not hold up the peer review process.

5. If the reviewer comments include a recommendation to cite specific previously published works, please review and evaluate these publications to determine whether they are relevant and should be cited. There is no requirement to cite these works unless the editor has indicated otherwise.

[Note: HTML markup is below. Please do not edit.]

Reviewers' comments:

Reviewer's Responses to Questions

**Comments to the Author**

1. Is the manuscript technically sound, and do the data support the conclusions?

The manuscript must describe a technically sound piece of scientific research with data that supports the conclusions. Experiments must have been conducted rigorously, with appropriate controls, replication, and sample sizes. The conclusions must be drawn appropriately based on the data presented.

Reviewer #1: No

Reviewer #2: Yes

**********

2. Has the statistical analysis been performed appropriately and rigorously?

Reviewer #1: No

Reviewer #2: No

**********

3. Have the authors made all data underlying the findings in their manuscript fully available?

The PLOS Data policy requires authors to make all data underlying the findings described in their manuscript fully available without restriction, with rare exception (please refer to the Data Availability Statement in the manuscript PDF file). The data should be provided as part of the manuscript or its supporting information, or deposited to a public repository. For example, in addition to summary statistics, the data points behind means, medians and variance measures should be available. If there are restrictions on publicly sharing data—e.g. participant privacy or use of data from a third party—those must be specified.

Reviewer #1: Yes

Reviewer #2: Yes

**********

4. Is the manuscript presented in an intelligible fashion and written in standard English?

PLOS ONE does not copyedit accepted manuscripts, so the language in submitted articles must be clear, correct, and unambiguous. Any typographical or grammatical errors should be corrected at revision, so please note any specific errors here.

Reviewer #1: Yes

Reviewer #2: Yes

**********

5. Review Comments to the Author

Please use the space provided to explain your answers to the questions above. You may also include additional comments for the author, including concerns about dual publication, research ethics, or publication ethics. (Please upload your review as an attachment if it exceeds 20,000 characters)

Reviewer #1: This is a somewhat dated analysis of the virological response to dual therapy (2DR) with nevirapine (NVP) and lamivudine (3TC) in people who previously achieved viral suppression on 3DR containing nevirapine and the results have limited clinical applicability. The rationale is that once people who can tolerate NVP are identified then NVP-based 2DR is a viable long-term option for these individuals. Although most people now use INSTI-based dual therapy in this setting NVP-based 2DR are still an alternative option for a minority of people who do not wish to change to INSTI.

I have several points that need to be addressed

Main points

1. This is essentially a mono-arm observational study which is sold as a clinical trial. I do not really see the need or advantage to call it a trial with a comparator “virtual control arm” that has 100% efficacy. Indeed, the procedure might be conservative, but strictly speaking because the control arm is only virtual with no simulated data being created, recommended tests (e.g. the Farrington -Manning score) cannot be performed in this study and the simple calculation of a proportion with a Clopper-Pearson 95% CI cannot be used to establish non-inferiority to the virtual arm. It would have been more informative to show it as a cohort with maximum 96 weeks follow-up and show the incidence of virological as well as treatment failure at different time-points. To this extent it is unclear why it was not possible to follow-up this cohort to more recent times. A 3-year estimate of the proportion still receiving the 2DR NVP-based regimen with HIV-RNA<50 copies/mL would have been a valuable information.

2. Indeed, being a maintenance study, the main outcome should be durability and not virological failure. Suggest that the authors use a time to treatment failure (TF) approach counting as events: i) virological failures, ii) all discontinuations regarding of the reason and iii) death.

3. Seven premature discontinuations have been excluded. There should be at least one TF analysis in which these are instead counted as events. At least the 4 discontinuations due to SAE and the single drug to drug interactions should be events. There were also 2 deaths which occurred during the study period (I assume these were among the 194 included in the analysis).

4. Using TF as primary endpoint with 7 failures and 201 participants the incidence rate is 3.5% with exact limits of (1.4-7.0%). Thus, even keeping the novel approach for a non-inferiority trial design, data clearly fail to show non-inferiority when using a more relevant clinical endpoint.

Minor points

1. Bottom of page 2. Occasional viral blips are described as HIV-RNA measurements of 50-200 cp/ml followed by an additional measurement of <50 cp/ml. This is not an accurate definition of blip. It should be a re-suppression to a value of <50 cp/ml but without any change in ART (certainly without a change of NVP).

2. Page 4. I would avoid expressions such as “median change was not significant” presumably linked to the use of a 5% threshold for the p-value. Authors should report in terms of strength of evidence against the null.

3. Page 5 Discussion. There is no reference to the last part of this sentence “Despite NVP having a low resistance barrier and requiring only a single mutation in the reverse transcriptase genome to develop resistance(15), DT with 3TC proved to be an efficient combination to achieve constant viral suppression”. In general, it seems out of place as the study evaluates maintenance of suppression not initial achievement.

Reviewer #2: This study presents an interesting multicenter non-inferiority trial investigating the efficacy of a switch strategy combining nevirapine and lamivudine in patients on cART with sustained HIV plasma viral load (pVL) <50 copies/mL for at least two years.

The Methods section is clearly described; however, the statistical analysis would benefit from a thorough review by a biostatistics expert.

The Results section is well written, although I have a few minor concerns:

- In the Introduction, the authors state that “NVP is the only NNRTI that does not negatively impact lipid levels.” However, the effect on lipid profiles was not evaluated in this study. Including such an analysis could strengthen the rationale for choosing a regimen with a lower resistance barrier compared to currently available dual therapies, particularly those based on integrase inhibitors (INIs).

- Darunavir/ritonavir/dolutegravir should not be considered a triple therapy.

The Discussion is well articulated, with an appropriate number of references. Moreover, the study limitations are clearly acknowledged.

**********

6. PLOS authors have the option to publish the peer review history of their article (what does this mean?). If published, this will include your full peer review and any attached files.

If you choose “no”, your identity will remain anonymous but your review may still be made public.

**Do you want your identity to be public for this peer review?**    For information about this choice, including consent withdrawal, please see our Privacy Policy.

Reviewer #1: No

Reviewer #2: No

**********

[NOTE: If reviewer comments were submitted as an attachment file, they will be attached to this email and accessible via the submission site. Please log into your account, locate the manuscript record, and check for the action link "View Attachments". If this link does not appear, there are no attachment files.]

To ensure your figures meet our technical requirements, please review our figure guidelines: https://journals.plos.org/plosone/s/figures

You may also use PLOS’s free figure tool, NAAS, to help you prepare publication quality figures: https://journals.plos.org/plosone/s/figures#loc-tools-for-figure-preparation.

NAAS will assess whether your figures meet our technical requirements by comparing each figure against our figure specifications.

# Author comment: Author response to Decision Letter 1

**DOI:** 10.1371/journal.pone.0351576.r003

13 May 2026

For the detailed responses to the editor and reviewers, we refer to the separately uploaded document.

Main points

1.This is essentially a mono-arm observational study which is sold as a clinical trial. I do not really see the need or advantage to call it a trial with a comparator “virtual control arm” that has 100% efficacy. Indeed, the procedure might be conservative, but strictly speaking because the control arm is only virtual with no simulated data being created, recommended tests (e.g. the Farrington -Manning score) cannot be performed in this study and the simple calculation of a proportion with a Clopper-Pearson 95% CI cannot be used to establish non-inferiority to the virtual arm. It would have been more informative to show it as a cohort with maximum 96 weeks follow-up and show the incidence of virological as well as treatment failure at different time-points. To this extent it is unclear why it was not possible to follow-up this cohort to more recent times. A 3-year estimate of the proportion still receiving the 2DR NVP-based regimen with HIV-RNA<50 copies/mL would have been a valuable information.

We appreciate the detailed comment of the reviewer. We have now simulated data for an ideal control group consisting of 194 patients with no observed virological failures, and we performed the Farrington-Manning-Test for non-inferiority of DT as suggested. We also applied the same approach for the post-study observation. We have decided not to simulate a virtual control group using the `rbinom` function from the R base package, since non-inferiority is demonstrated in all cases where X0 > 0. In addition, an arbitrary yet justified assumption about the exact failure rate in the control group would need to be made in order to obtain a distribution.

While we agree that a 3-year estimate of the proportion of participants with HIV-RNA <50 cp/ml would further enhance the study, the multicenter design makes extended data collection challenging. Consequently, the study period was defined as 48 weeks following FDA guidelines for HIV maintenance therapy, supplemented by a post-study observation of up to 12 months. However, we additionally re-contacted the other centers to supplement further results, ensuring that there were no missing data during the 12-month post-study observation period. Regarding an extended follow-up, we are planning follow-up studies to assess long-term safety and the impact of this dual therapy on the viral reservoir, as noted at the end of the discussion.

2.Indeed, being a maintenance study, the main outcome should be durability and not virological failure. Suggest that the authors use a time to treatment failure (TF) approach counting as events: i) virological failures, ii) all discontinuations regarding of the reason and iii) death.

Focusing on durability is an interesting consideration. However, our study was originally designed in accordance with FDA guidelines, with virological failure as the primary endpoint. Since our study was designed in alignment with other HIV non-inferiority trials, the proposed approach (TF) is not feasible. In the assessment of HIV maintenance therapy, the primary focus is on virological suppression; other events or discontinuations are counted only if patients are not virologically suppressed. All discontinuations and deaths were carefully reviewed for any potential association with the dual therapy, and no such correlation was observed. We have updated the Methods section to specify that treatment failure refers to virological failure.

3. Seven premature discontinuations have been excluded. There should be at least one TF analysis in which these are instead counted as events. At least the 4 discontinuations due to SAE and the single drug to drug interactions should be events. There were also 2 deaths which occurred during the study period (I assume these were among the 194 included in the analysis).

In line with the study design based on the guidelines mentioned above, participants were required to complete the 48-week period to be included in the final analysis (i.e., 194 patients; Fig. 1). The deaths would only have been included, if virological failure occurred during the study period among these patients. The circumstances of the death events are described in the Results section.

4. Using TF as primary endpoint with 7 failures and 201 participants the incidence rate is 3.5% with exact limits of (1.4-7.0%). Thus, even keeping the novel approach for a non-inferiority trial design, data clearly fail to show non-inferiority when using a more relevant clinical endpoint.

The FDA-defined non-inferiority margin of 4% was used to demonstrate non-inferiority specifically for the primary endpoint of virological failure. Using the TF approach would make it challenging to determine a valid non-inferiority margin, particularly when comparing the results to a perfect virtual control group with no events. If the TF approach was applied, a different study design with a real control group, for example a randomized controlled trial, would be more appropriate.

Minor points:

1.Bottom of page 2. Occasional viral blips are described as HIV-RNA measurements of 50-200 cp/ml followed by an additional measurement of <50 cp/ml. This is not an accurate definition of blip. It should be a re-suppression to a value of <50 cp/ml but without any change in ART (certainly without a change of NVP).

Thank you for your input; we have revised the sentence as suggested.

2. Page 4. I would avoid expressions such as “median change was not significant” presumably linked to the use of a 5% threshold for the p-value. Authors should report in terms of strength of evidence against the null.

We appreciate this comment. In the manuscript, we reported the exact p-value as a measure of evidence against the null hypothesis. For further clarification, we have now explicitly specified the null hypothesis.

3. Page 5 Discussion. There is no reference to the last part of this sentence “Despite NVP having a low resistance barrier and requiring only a single mutation in the reverse transcriptase genome to develop resistance(15), DT with 3TC proved to be an efficient combination to achieve constant viral suppression”. In general, it seems out of place as the study evaluates maintenance of suppression not initial achievement.

We have rephrased the final part of the sentence. Since the statement is based on our study, no external reference is necessary in our opinion.

Reviewer 2

This study presents an interesting multicenter non-inferiority trial investigating the efficacy of a switch strategy combining nevirapine and lamivudine in patients on cART with sustained HIV plasma viral load (pVL) <50 copies/mL for at least two years. The Methods section is clearly described; however, the statistical analysis would benefit from a thorough review by a biostatistics expert. The Results section is well written, although I have a few minor concerns:

- In the Introduction, the authors state that “NVP is the only NNRTI that does not negatively impact lipid levels.” However, the effect on lipid profiles was not evaluated in this study. Including such an analysis could strengthen the rationale for choosing a regimen with a lower resistance barrier compared to currently available dual therapies, particularly those based on integrase inhibitors (INIs).

Thank you for your valuable review and suggestions. Considering your suggestions, those of Reviewer 1 and in addition to the supplementary analysis, the manuscript has undergone a thorough review by a statistician (see Acknowledgements).

Furthermore, we agree with the reviewer that analyzing changes in the lipid profile could provide an additional reason for choosing this dual therapy. However, lipid parameters were available only from certain centers (i.e. below 50% of the whole population); therefore, any conclusions drawn from these data would have limited informative value. However, information on this point is available from the literature which is cited, and it is mentioned in the manuscript as a limitation. We now explicitly refer to lipid levels as an example.

-Darunavir/ritonavir/dolutegravir should not be considered a triple therapy.

Thank you for the clarification. We have revised the sentence accordingly.

Attachment Submitted filename: Response to Reviewers PLOS ONE.docx

# Editor report: Decision Letter 1

**DOI:** 10.1371/journal.pone.0351576.r004

**Authors:** Giuseppe De Socio

28 May 2026

A novel design using a virtual control group to evaluate non-inferiority of nevirapine and lamivudine dual maintenance in HIV therapy

PONE-D-25-54404R1

Dear Dr. Suter,

We’re pleased to inform you that your manuscript has been judged scientifically suitable for publication and will be formally accepted for publication once it meets all outstanding technical requirements.

Within one week, you’ll receive an e-mail detailing the required amendments. When these have been addressed, you’ll receive a formal acceptance letter and your manuscript will be scheduled for publication.

An invoice will be generated when your article is formally accepted. Please note, if your institution has a publishing partnership with PLOS and your article meets the relevant criteria, all or part of your publication costs will be covered. Please make sure your user information is up-to-date by logging into Editorial Manager at Editorial Manager® and clicking the ‘Update My Information' link at the top of the page. For questions related to billing, please contact billing support.

If your institution or institutions have a press office, please notify them about your upcoming paper to help maximize its impact. If they’ll be preparing press materials, please inform our press team as soon as possible -- no later than 48 hours after receiving the formal acceptance. Your manuscript will remain under strict press embargo until 2 pm Eastern Time on the date of publication. For more information, please contact onepress@plos.org.

Kind regards,

Giuseppe Vittorio De Socio, MD, PhD

Academic Editor

PLOS One

Additional Editor Comments (optional):

Reviewers' comments:

# Editor report: Acceptance letter

**DOI:** 10.1371/journal.pone.0351576.r005

**Authors:** Giuseppe De Socio

PONE-D-25-54404R1

PLOS One

Dear Dr. Suter,

I'm pleased to inform you that your manuscript has been deemed suitable for publication in PLOS One. Congratulations! Your manuscript is now being handed over to our production team.

At this stage, our production department will prepare your paper for publication. This includes ensuring the following:

* All references, tables, and figures are properly cited

* All relevant supporting information is included in the manuscript submission,

* There are no issues that prevent the paper from being properly typeset

You will receive further instructions from the production team, including instructions on how to review your proof when it is ready. Please keep in mind that we are working through a large volume of accepted articles, so please give us a few days to review your paper and let you know the next and final steps.

Lastly, if your institution or institutions have a press office, please let them know about your upcoming paper now to help maximize its impact. If they'll be preparing press materials, please inform our press team within the next 48 hours. Your manuscript will remain under strict press embargo until 2 pm Eastern Time on the date of publication. For more information, please contact onepress@plos.org.

You will receive an invoice from PLOS for your publication fee after your manuscript has reached the completed accept phase. If you receive an email requesting payment before acceptance or for any other service, this may be a phishing scheme. Learn how to identify phishing emails and protect your accounts at https://explore.plos.org/phishing.

If we can help with anything else, please email us at customercare@plos.org.

Thank you for submitting your work to PLOS ONE and supporting open access.

Kind regards,

PLOS ONE Editorial Office Staff

on behalf of

prof. Giuseppe Vittorio De Socio

Academic Editor

PLOS One
